# Exploring the biological function of immune cell-related genes in human immunodeficiency virus (HIV)-1 infection based on weighted gene co-expression network analysis (WGCNA)

**DOI:** 10.1186/s12920-022-01357-y

**Published:** 2022-09-19

**Authors:** Ruojing Bai, Zhen Li, Shiyun Lv, Wei Hua, Lili Dai, Hao Wu

**Affiliations:** 1 Beijing Key Laboratory for HIV/AIDS Research, Sino-French Joint Laboratory for Research On Humoral Immune Response to HIV Infection, Clinical and Research Center for Infectious Diseases, Beijing Youan Hospital, Capital Medical University, Beijing, China; 2 Travel Clinic, Center for Infectious Diseases, Beijing Youan Hospital, Capital Medical University, Beijing, 100069 China

**Keywords:** AIDS, Gene co-expression network analysis, ceRNA network, HIV-1

## Abstract

**Background:**

Acquired immunodeficiency syndrome (AIDS) is a chronic infectious disease characterized by consistent immune dysfunction. The objective of this study is to determine whether immune cell-related genes can be used as biomarkers for the occurrence of AIDS and potential molecular mechanisms.

**Methods:**

A weighted gene co-expression network analysis was performed using the GSE6740 dataset from the Gene Expression Synthesis Database to identify the Hub gene, which contained microarray data from HIV-1 positive (HIV-1^+^) and HIV-1 negative (HIV-1^−^) individuals. The HIV-1^+^-related differentially expressed genes were then identified using the *limma* package. Subsequently, the characteristic immune cell-related genes were identified as diagnostic biomarkers for HIV-1^+^ using the random forest model (RF), support vector machine model, and generalized linear model.

**Results:**

MEdarkgreen exhibited the strongest correlation with HIV clinical features of any of these modules. As the best model for diagnosing HIV-1^±^, RF was used to select four critical immune cell-related genes, namely, ARRB1, DPEP2, LTBP3, and RGCC, and a nomogram model was created to predict the occurrence of HIV-1 infection based on four key immune cell-related genes. Diagnostic genes were shown to be engaged in immune-related pathways, suggesting that immunological molecules, immune cells, and immune pathways all have a role in HIV-1 infection. The CTD database was explored for prospective medications or molecular compounds that might be utilized to treat HIV-1^+^ patients. = Moreover, in HIV-1^+^ individuals, the ceRNA network revealed that ARRB1, DPEP2, LTBP3, and RGCC could be regulated by lncRNAs through the corresponding miRNAs. Ultimately, RT-PCR results from clinical blood samples demonstrated that the four diagnostic genes were significantly downregulated in HIV-1^+^ patients.

**Conclusion:**

We screened four immune cell-related genes, ARRB1, DPEP2, LTBP3, and RGCC, which may be considered as the diagnostic markers for HIV-1/AIDS. Our findings reveal that immune related genes and pathways involved in HIV-1 pathogenesis were regulated on both genetic and epigenetic levels by constructing a ceRNA network associated with lncRNA.

**Supplementary Information:**

The online version contains supplementary material available at 10.1186/s12920-022-01357-y.

## Background

### Background of Acquired Immunodeficiency Syndrome (AIDS)

Acquired immunodeficiency syndrome (AIDS) refers to a chronic infectious disease characterized by human immunodeficiency virus (HIV)-1 infection [1]. One of the indications of infection is the continuous destruction of CD4 + T cells, which can lead to increased immune deficiency and mortality [2]. According to data from UNAIDS, approximately 37.9 million people worldwide are infected with HIV, with nearly 770,000 people dying as a result of HIV-related diseases [3, 4]. Integrase strand transfer inhibitors (INSTI), such as Raltegravir (RAL), Bictegravir (BIC), Dolutegravir (DTG), and Elvitegravir (EVG), are currently approved for the treatment of HIV-1 infection [5]. Despite their ability to reduce the incidence and mortality of AIDS by inhibiting viral replication and controlling infection, these drugs are still unable to cure the disease, HIV-1 will be reactivated immediately to destroy immune system if treatment is stopped.

### Research progress on immunity and HIV/AIDS infection

The immune system is crucial in the pathogenesis of HIV infection and AIDS [6–8]. It has recently been reported that dendritic cells (DC) play a dual role in the transmission and elimination of HIV-1 infection. On the other hand, DC can improve the effectiveness of the preventive HIV-1 vaccine by generating pro-inflammatory cytokines and enhancing T cell activation [9]. Besides, dendritic cells in lymphoid organs can activate resting memory T cells, which could lead to the reactivation of HIV-1 transmission [10]. In the case of HIV-1 infection, the expression of PD-1 on virus-specific T cells is a crucial indicator of failure, which is linked to AIDS progression [11]. Th17 cells maintain the mucosal immune response to resist HIV-1 invasion by secreting inflammatory factors and antimicrobial peptides and recruiting neutrophils [12]. Increased natural killer cell (NK) activity implies high levels of cytotoxicity, IFN-γ, and chemokines (CCL3, CCL4, and CCL5), which is linked to anti-HIV-1 infection and delayed AIDS progression [13]. To the best of our knowledge, there have been few reports on whether immune cell-related genes can be applied as biomarkers for HIV-positive (HIV-1^+^).

### Our research results and significance

Therefore, the purpose of this study is to reveal the potential molecular mechanism of immune cell-related genes as HIV-1^+^ biomarkers, providing a novel idea to the diagnosis and treatment of HIV-1^+^.

## Methods

### Dataset download

The original gene expression profile file in the GSE6740 dataset was downloaded from the NCBI GEO database (https://www.ncbi.nlm.nih.gov/). The GSE6740 dataset, obtained from Affymetrix Human Genome U133 A Array [HG-U133 A] platform, is mainly comprised of 10 HIV-1-negative (HIV-1^−^) samples and 30 HIV-1^+^ samples for further analysis [14].

### Weighted gene co-expression network analysis (WGCNA)

The expression profile data of all genes in the GSE6740 dataset were used to create the gene co-expression network and identify the key modules using the WGCNA program in the R package [15]. Besides, HIV-1^+^, HIV-1^−^, CD4 cells, and CD8 cells were considered clinical traits for WGCNA analysis. To begin, cluster analysis was applied to the samples in the GSE6740 dataset to identify any outliers. The four clinical characters, including HIV-1^+^, HIV-1^−^, CD4, and CD8 cells, were then added to the clustering map to construct sample clustering and clinical character heat map. For the construction of scale-free co-expression networks, the function pickSoft Threshold was adopted to determine the optimal soft threshold power as 14 (scale-free R^2^ = 0.85). The adjacency matrix was then converted into a topological overlap matrix (TOM). The minimum number of genes per gene module was set to 30 using the hybrid dynamic tree-cutting algorithm, and MEDissThres was set to 0.3 to merge similar modules. A calculation was undertaken to investigate the association between gene modules and HIV-1 clinical characteristics. In this investigation, the modules having the highest connection with these four clinical criteria were designated as important modules. Among them, the genes satisfying the conditions | Module membership (MM) |> 0.8 and | Gene significance (GS) |> 0.2 were taken as hub genes.

### Screening of differentially expressed genes

The gene expression matrix of 40 samples was constructed by standardizing the expression data in the dataset GSE6740. The differential expression of 30 HIV-1^+^ and 10 HIV-1^−^ samples was evaluated using the R package *limma*. The screening threshold for differentially expressed genes (DEGs) was P-value 0.5, and the candidate genes were determined using Venn analysis by crossing the differential genes and Hub genes.

### Enrichment analysis of candidate genes

A gene-GO network was constructed to perform gene ontology (GO) enrichment analysis on candidate genes by inputting the candidate genes into ClueGO and CluePedia for analysis. ClueGo and CluePedia were both done using Cytoscape 3.8.0 software, with the default settings for GO terms/path selection options used for optimal enrichment visualization.

### Construction and evaluation of random forest model (RF), support vector machine model (SVM), and generalized linear model (GLM)

The caret in the R package was used to construct a random forest model (RF), support vector machine model (SVM), and generalized linear model (GLM) based on the expression of candidate genes in the dataset GSE6740 and sample grouping, with HIV-1^+^ diagnosis or not as a response variable and candidate genes as explanatory variables. To obtain the best model based on the data set, the three previously mentioned models were analyzed using the interpretation characteristics of the DALEX package in R, while the plot function was used to draw the cumulative residual distribution map and the box diagram distribution map, respectively. Meanwhile, an analysis of the relative importance of variables in different models was carried out, with the most relevant explanatory variables chosen for the receiver operating characteristic (ROC) analysis to explore the distinct ability of these genes in GSE6740.

### Construction and evaluation of nomogram

A map model for clinical applications for gene diagnostic markers was created using the *rms* package. “Score” refers to the scores of the following corresponding factors under different conditions, and “total score” indicates the sum of all the above factors. The calibration curve was then generated using the calibration function in the *rms* package, and the decision curve (DCA) was drawn using the *rmda* package, to evaluate the clinical significance of the nomogram. Furthermore, the nomogram model was evaluated for its clinical application in analyzing the clinical impact curve based on the DCA curve.

### Diagnostic marker-drug interaction network analysis

To provide detailed information such as chemical gene/protein interactions, chemical diseases, and gene-disease relationships, the comparative toxicological genomics database (CTD, http://ctdbase.org/) was used [16]. Diagnostic markers were obtained from the CTD database in order to get potential medications or molecular compounds for HIV-1^+^ therapy, and the diagnostic marker-molecular compound interaction network was visualized using Cytoscape software.

### Construction of CeRNA network

The ENCORI database (http://starbase.sysu.edu.cn/tutorial.php) is public online platform for studying RNA interactions [17]. In this study, the ENCORI database was used to predict the miRNAs associated with diagnostic markers (mRNAs) and the lncRNAs associated with miRNAs, as well as to investigate the correlation between diagnostic genes and miRNAs and between miRNA and lncRNA (|cor|> 0.3, *P* < 0.05), in order to develop a lncRNA-miRNA-mRNA regulatory network in HIV-1^+^. The potential binding sites of miRNA sequences and mRNAs, as well as lncRNA sequences and miRNAs, were demonstrated in the ENCORI database based on the above interactions between miRNAs and mRNAs, and miRNAs and lncRNAs. Following that, the ceRNA network was built with Cytoscape software utilizing the mRNA-miRNA and miRNA-lncRNA interaction pairs identified in the previous analysis.

### RNA Extraction and RT-PCR Detection

A total of 10 HIV-1^−^ and 10 HIV-1^+^ whole blood samples were collected from the sexually transmitted disease (STD) and AIDS Clinic, Beijing Youan Hospital, Capital Medical University. The study protocol was approved by the ethics committee of Beijing You'an Hospital of Capital Medical University (LL-2019-038-K). All participants provided written informed consent.

Total RNA was extracted from blood using Nuclezol LS RNA Isolation Reagent (ABP Biosciences Inc), and the quality of the RNA was determined using a nucleic acid-protein instrument. RNA was reverse transcribed into cDNA in line with the instructions of SureScript-First-strand-cDNA-synthesis-kit (GeneCopoeia), and BlazeTaq SYBR Green qPCR mix 2.0 (GeneCopoeia) was adopted to detect the relative expression of the target gene. The specific primers of the gene include DPEP2 forward primer 5′- ACCTGACGCTCACCCACACCT -3′; DPEP2 reverse primer 5′- GGCCCCATCATAATCTCCACC -3′; RGCC forward primer 5′- TCTCCAACAGA CTCTACCCCAG CAT-3′; RGCC reverse primer 5′- GTTTTGTCAAGATCAGCAA TGA -3′; ARRB1forward primer 5′- AACTGCCCTTCACCCTAAT CAT-3′; ARRB1 reverse primer 5′- TTCCTCCTTGTCATCCTTC -3′; LTBP3 forward primer 5′- ACA TCGTCAACTACGGCATCC CAT-3′; LTBP3 reverse primer 5′- CTCGTCCACGT CCATCTCTTC -3′; GAPDH forward primer 5′- CCTTCCGTGTTCCTACCCCCA T-3′; GAPDH reverse primer 5′- GCCCAAGATGCCCTTCAGT -3′. The CT values of the genes were counted, and GAPDH was treated as the internal reference gene to calculate the relative expression level of the target gene using the 2^−△△Ct^ method.

### Statistical analysis

All analyses were conducted with R version 3.5 (https://www.r-project.org/) and its several open packages. The two-sided *P* value < 0.05 was considered statistically significant. Other used visual packages included WGCNA, heatmap, and DALEX.

## Results

### ***Screening of modules highly associated with immune cells in HIV-1***^+^

**Fig. 1:**
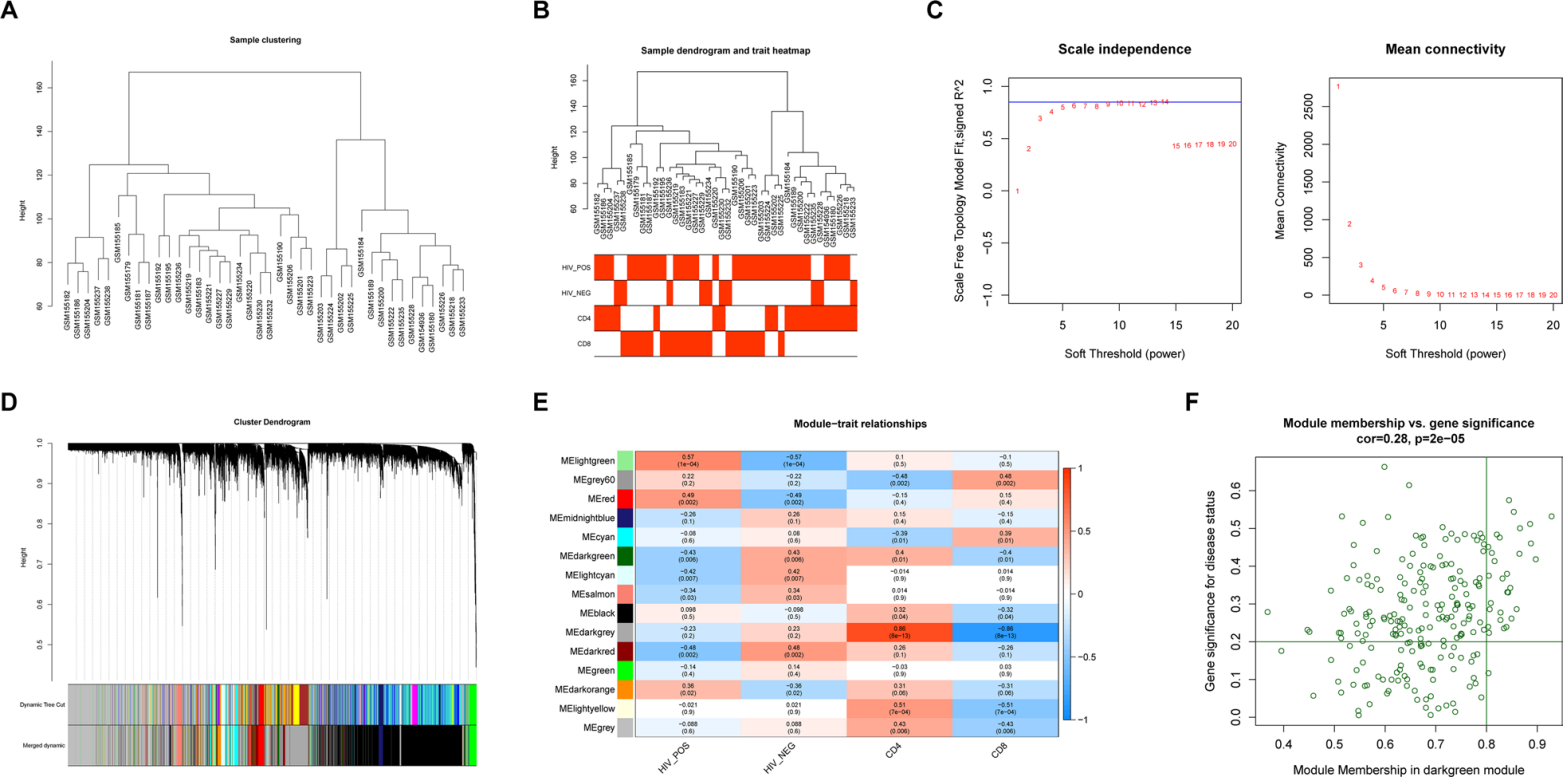
Screening of highly related modules. **A** The clustering dendrogram of HIV-1^±^ samples to detect outliers. **B** Cluster tree of 10 HIV-1^−^ and 30 HIV-1^+^ samples in the GSE6740 dataset. The color band underneath the tree indicates the numeric values of the tissue traits. **C** The scale-free fit index for soft-thresholding powers. The soft-thresholding power in the WGCNA was determined based on a scale-free R2 (R2 = 0.85). The left panel shows the scale-free fit index (y-axis) as a function of the soft-thresholding power (x-axis). The right panel displays the mean connectivity (degree, y-axis) as a function of the soft-thresholding power (x-axis). **D** Dendrogram of all genes clustered based on the measurement of dissimilarity. **E** A heatmap showing the correlation between the gene module and clinical traits: Each row corresponds to a module eigengene and each column to a trait. Each cell contains the corresponding correlation and p-value. The table is color-coded by correlation according to the color legend, which decreased in size from red to blue. **F** Scatter plot of module eigengenes related to HIV-1^+^ in the darkgreen module

The whole flowchart of the study is shown in Additional file 1: Figure S1. WGCNA was run on 10 HIV-1^−^ samples and 30 HIV-1^+^ samples in the GSE6740 dataset to determine the essential modules most closely connected to the clinical characteristics of HIV-1^+^. Prior to WGCNA, cluster analysis was conducted to ensure that there were no outlier samples in the data set (Fig. 1A). The clinical information of HIV-1, such as HIV-1^+^, HIV-1^−^, CD4 cells, and CD8 cells, was retrieved from the GSE6740 dataset and included in the clustering diagram (Fig. 1B). The soft threshold power was set to 14 (scale-free R^2^ = 0.85), conforming to the scale-free distribution to the maximum extent (Fig. 1C), and the dynamic shear tree algorithm was applied to determine 15 modules (Fig. 1D). A detailed examination of the module-clinical feature correlation heat map revealed that the MEdarkgreen module had the most significant association with the four clinical features (Fig. 1E, |correlation coefficient|> 0.4, *P* < 0.01), hence it was designated as the key module. The MEdarkgreen module produced 225 genes, 19 of which met the criteria (|MM|> 0.8 and |GS |> 0.2) and were identified as Hub genes (Fig. 1F).

### ***Screening candidate genes for HIV-1***^+^

**Fig. 2:**
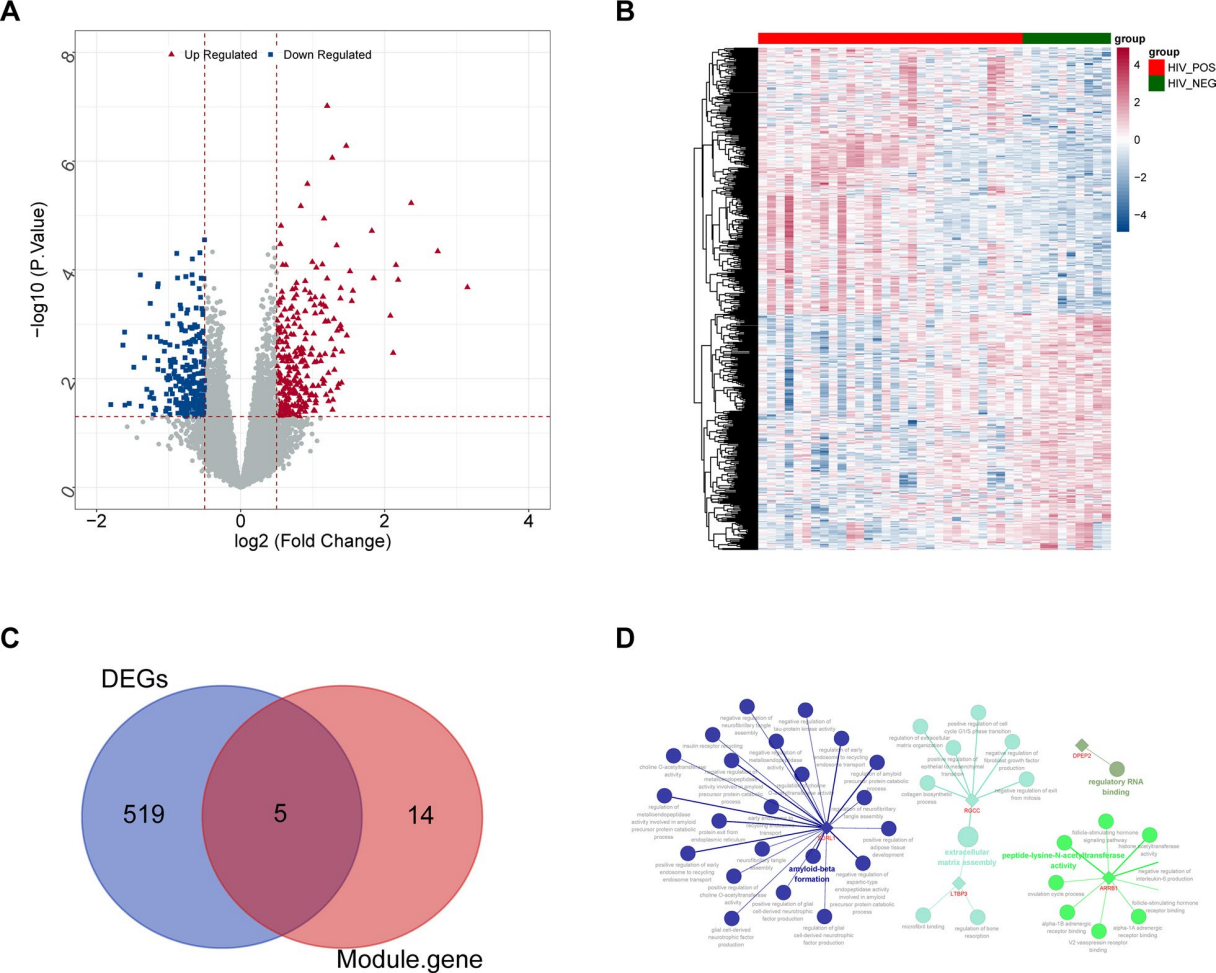
Identification of DEGs between 30 HIV-1^+^ and 10 HIV-1^−^ samples **A**, **B**. volcano plot **A** and Heatmap **B** presented the expression of DEGs. **C** The Venn diagram for key genes. **D** The functional annotation analysis of the five candidate genes was performed by ClueGO and CluePedia. The candidate genes and enrichment terms constitute a regulatory network

The *limma* package was used to evaluate the DEGs between HIV-1^+^ and HIV-1^−^ samples in the GSE6740 dataset, with *P* < 0.5 chosen as the screening threshold. A total of 524 DEGs in HIV-1^+^ samples were evaluated in contrast to HIV-1^−^ samples, comprising 278 up-regulated genes and 246 down-regulated genes (Additional file 2: Table S1). Figure 2A and B represent these DEGs in the form of volcanic and thermal maps. Subsequently, these differential genes were overlapped with the hub genes in the MEdarkgreen module to determine five candidate genes (SORL1, DPEP2, RGCC, ARRB1, and LTBP3) (Fig. 2C). Furthermore, the ClueGO/CluePedia plug-in in Cytoscape software was applied to explore the GO of candidate genes (Fig. 2D, Additional file 2: Table S2). SORL 1 was shown to be involved in 22 important biological processes, including early endosome to recycling endosome transport (GO: 0,061,502), choline O-acetyltransferase activity regulation (GO: 1,902,769), and positive regulation of adipose tissue development (GO: 1,904,179). Amyloid precursor protein catabolic process was associated with protein exit from the endoplasmic reticulum (GO: 0,032,527), positive regulation of glial cell-derived neurotrophic factor production (GO: 1,900,168), regulation of amyloid precursor protein catabolic process (GO: 1,902,991), and negative regulation of metalloendopeptidase activity (GO: 1,902,963). Furthermore, the follicle-stimulating hormone signaling pathway was discovered to be linked to ARRB1, histone acetyltransferase activity (GO: 0,004,402), the ovulation cycle process (GO: 0,022,602), and the negative regulation of interleukin-6 production (GO: 0,032,715), (GO: 0,042,699), and DPEP2's molecular function was primarily involved in regulating RNA binding (GO: 0,061,980). In addition, RGCC was shown to be involved in the negative regulation of fibroblast growth factor synthesis, and both RGCC and LTBP3 have been connected to the regulation of extracellular matrix formation (GO: 0,085,029) during HIV-1 infection.

### ***Screening of HIV-1***^+^***diagnostic markers***

**Fig. 3:**
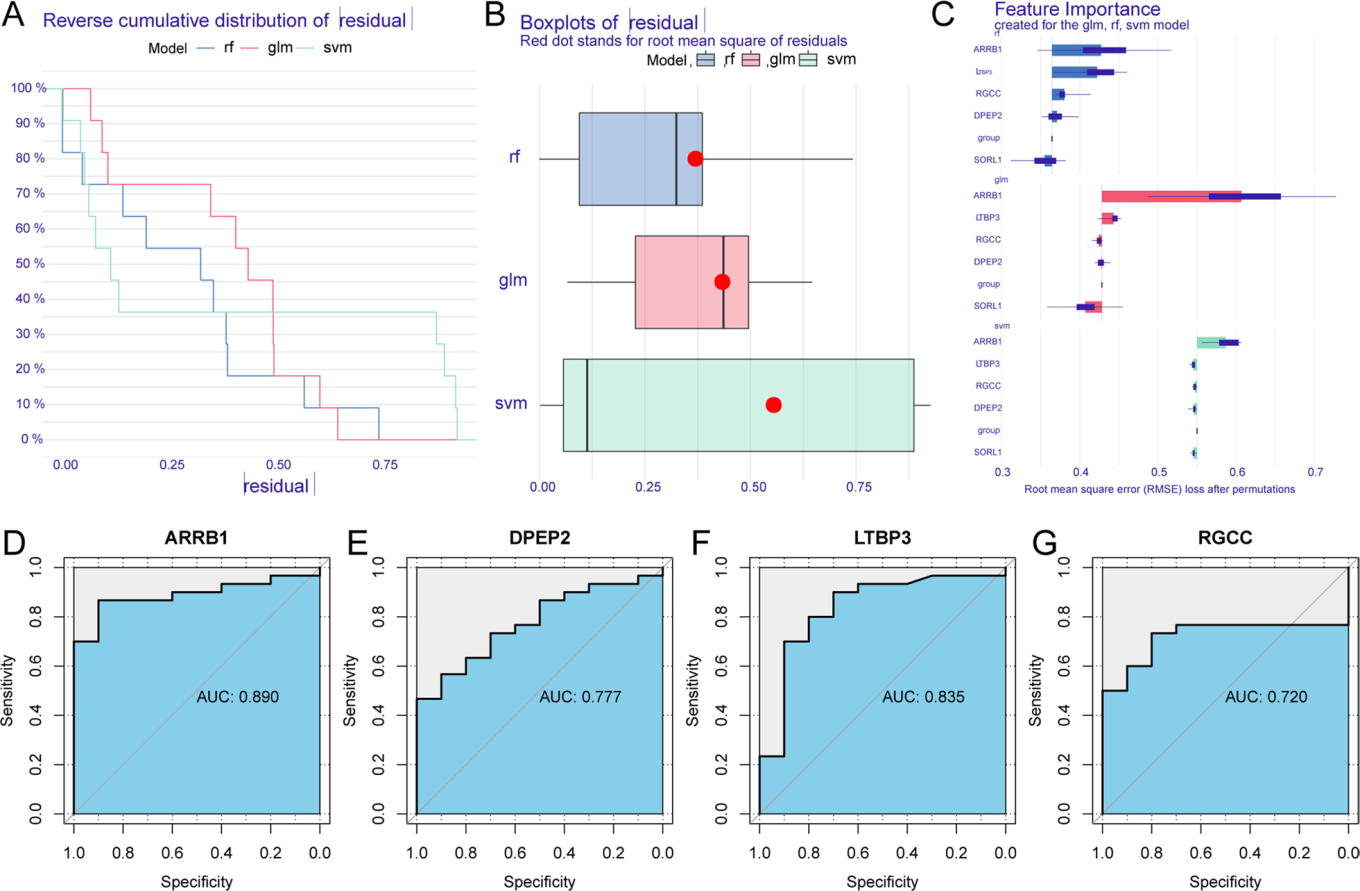
Construction and assessment of RF, GLM, and SVM model. **A** Cumulative residual distribution map of the sample. **B** Boxplots of the residuals of the sample. Red dot stands for root mean square of residuals. **C** The importance of the variables in RF, GLM, and SVM model

To further screen the genes with diagnostic value for AIDS, the GSE6740 dataset was used to establish a random forest model (RF), support vector machine model (SVM), and generalized linear model (GLM). These three models were then submitted to an explanatory analysis in R using the DALEX package, and the residual distribution map was plotted to identify the optimum model. The RF model, as illustrated in Fig. 3A and B, is considered to be the best match for the minimum sample residue. The four factors in the RF model, including DPEP2, RGCC, ARRB1, and LTBP3, have a considerable influence on the projected value of the response variable (Fig. 3C). The areas under the ROC curve of which were 0.777, 0.720, 0.890, 0.835, respectively (Fig. 3D–G), indicating that these genes had an excellent separating capacity between HIV-1- and HIV-1 + samples. Therefore, these four genes were taken as diagnostic markers for HIV-1^+^ to carry out further analysis.

### Construction and evaluation of a four diagnostic marker-based nomogram model

**Fig. 4:**
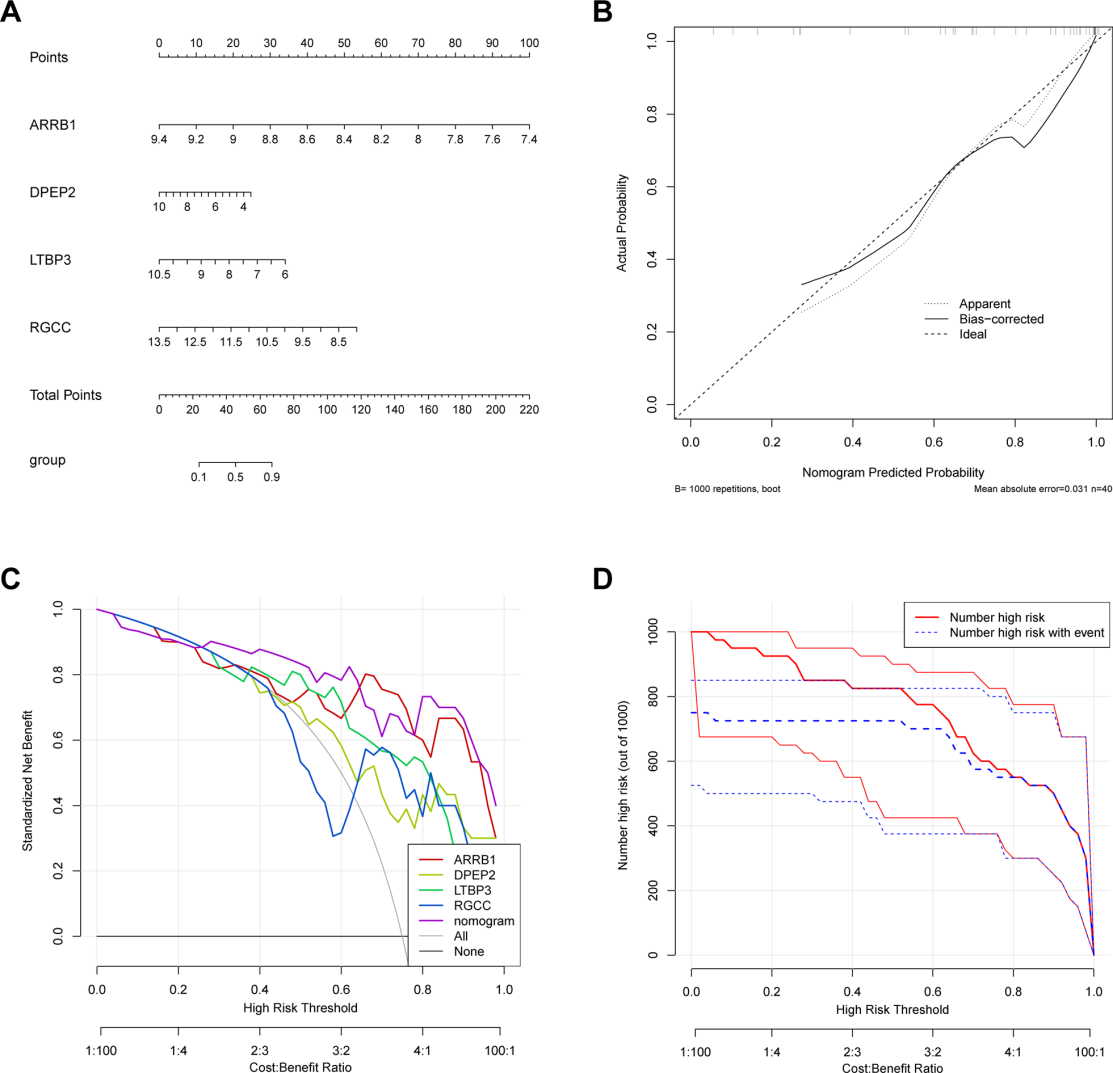
Construction and evaluation of the nomogram model based on the four diagnostic variables from the GSE6740 dataset. **A** Nomogram predicting the incidence of HIV-1^+^. The total points projected on the bottom scales indicate the incidence of HIV-1^+^. **B** The calibration curve revealed the predictiveness of the nomogram model. **C** The DCA curve evaluated the clinical value of the nomogram model. **D** The clinical impact curve was used to assess the clinical impact of the nomogram model

To enhance clinical prediction for HIV-1^+^, a nomogram model based on DPEP2, RGCC, ARRB1, and LTBP3 was constructed using the *rms* package (Fig. 4A), and the calibration curve (Fig. 4B) was established using the calibration function in the *rms* package to assess the nomogram model's predictive capacity. The calibration curve shows that the difference between the actual risk of HIV-1 infection and the projected risk is insignificant, implying that the nomogram model's prediction is highly accurate for HIV-1 infection diagnosis. As revealed by decision-making curve analysis (DCA), the “nomogram” curve is higher than the gray line, the “ARRB1” curve, the “DPEP2” curve, the “LTBP3” curve, and the “RGCC” curve. The x-axis in DCA represents forecast probability, while the y-axis represents net income. The oblique red line indicates that the nomogram model could benefit those patients with high-risk thresholds of 0 to 1, with the clinical benefit of the nomogram model outweighing that of the “ARRB1”, “DPEP2”, “LTBP3”, and “RGCC” curves, which suggested that the diagnostic value of the nomogram model for predicting HIV-1 infection (Fig. 4C). In addition, the clinical impact curve was further evaluated based on the DCA curve to determine the clinical effect of the “nomogram” model more intuitively. It was found that from 0 to 1, the “Number high risk” curve was close to the “Number high risk with the event” curve under the high-risk threshold, indicating the excellent ability of the “nomogram” model to predict HIV-1 infection (Fig. 4D). These findings also suggest that the four genes DPEP2, RGCC, ARRB1, and LTBP3 may play an important role in HIV-1 infection.

### Construction of diagnostic gene–drug interaction network

**Fig. 5:**
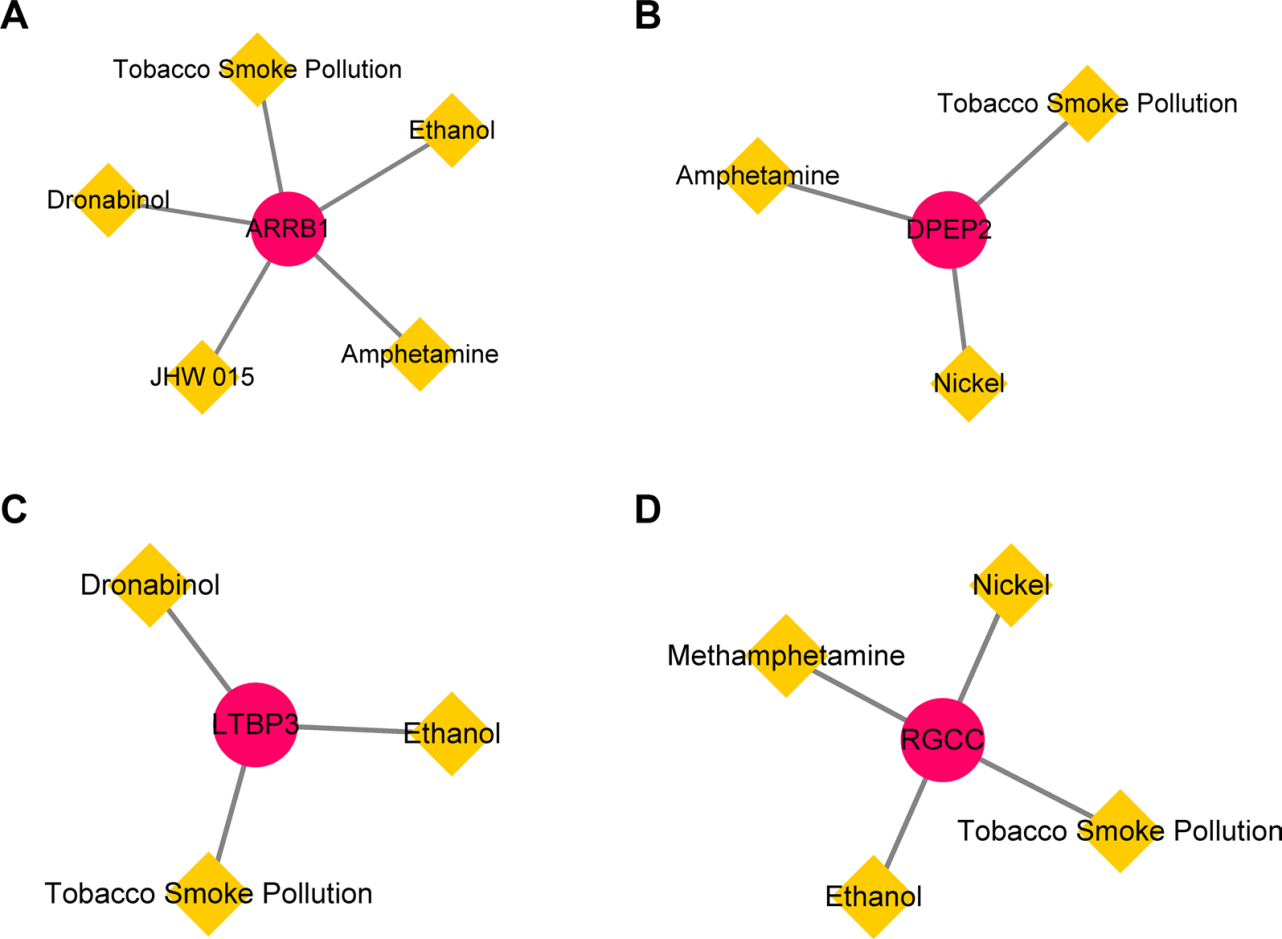
Drug-gene interactions network with chemotherapeutic drugs and four diagnostic genes was constructed using the CTD database. **A**–**D** The interaction between existing chemotherapeutic drugs and the diagnostic genes. **A** ARRB1. **B** DPEP2. **C** LTBP3. **D** RGCC

The potential drugs or molecular compounds (Additional file 2: Table S3) of diagnostic genes DPEP2, RGCC, ARRB1, and LTBP3 for HIV-1^+^ treatment were searched in the CTD database. A diagnostic gene-drug interaction network was created and visualized using Cytoscape to determine the interaction between diagnostic genes and existing HIV-1 medications (Fig. 5). During the treatment of HIV-1^+^, multiple drugs can affect the expression of these four diagnostic genes.

### Construction of CeRNA network

**Fig. 6:**
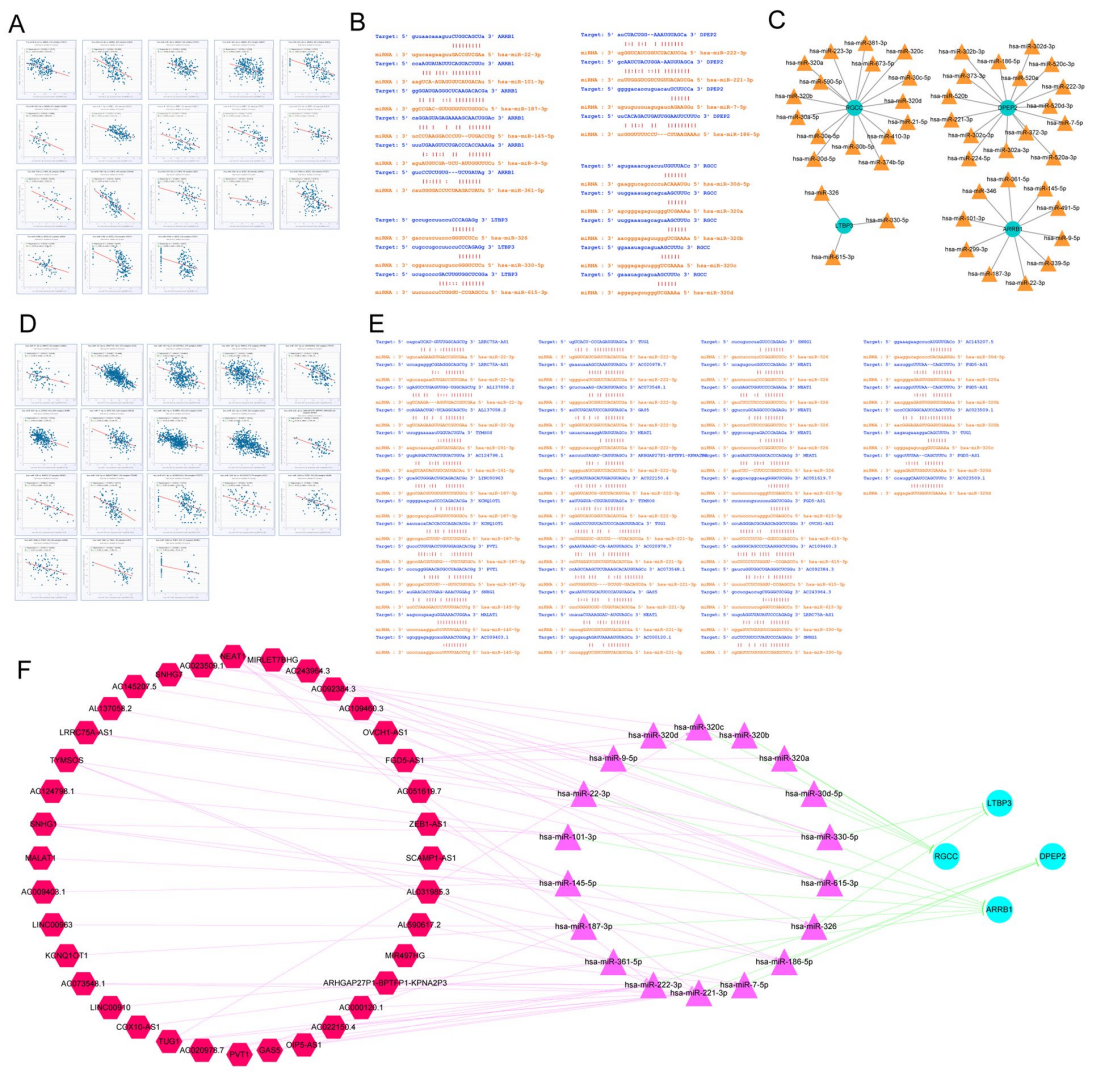
Construction of CeRNA network. **A** Diagnostic gene negative correlation miRNA screening. **B** The potential binding sites of miRNA to diagnostic genes. **C** The miRNA-diagnostic gene interaction networks, the yellow triangle represents miRNA and the aquamarine blue circle represents mRNA. **D** miRNA and lncRNA with the greatest correlation. **E** The potential binding sites of lncRNA sequence to miRNA. **F** ceRNA network, red hexagon indicates lncRNA, the purple triangle indicates miRNA and the light blue circle indicates diagnostic gene

**Fig. 7:**
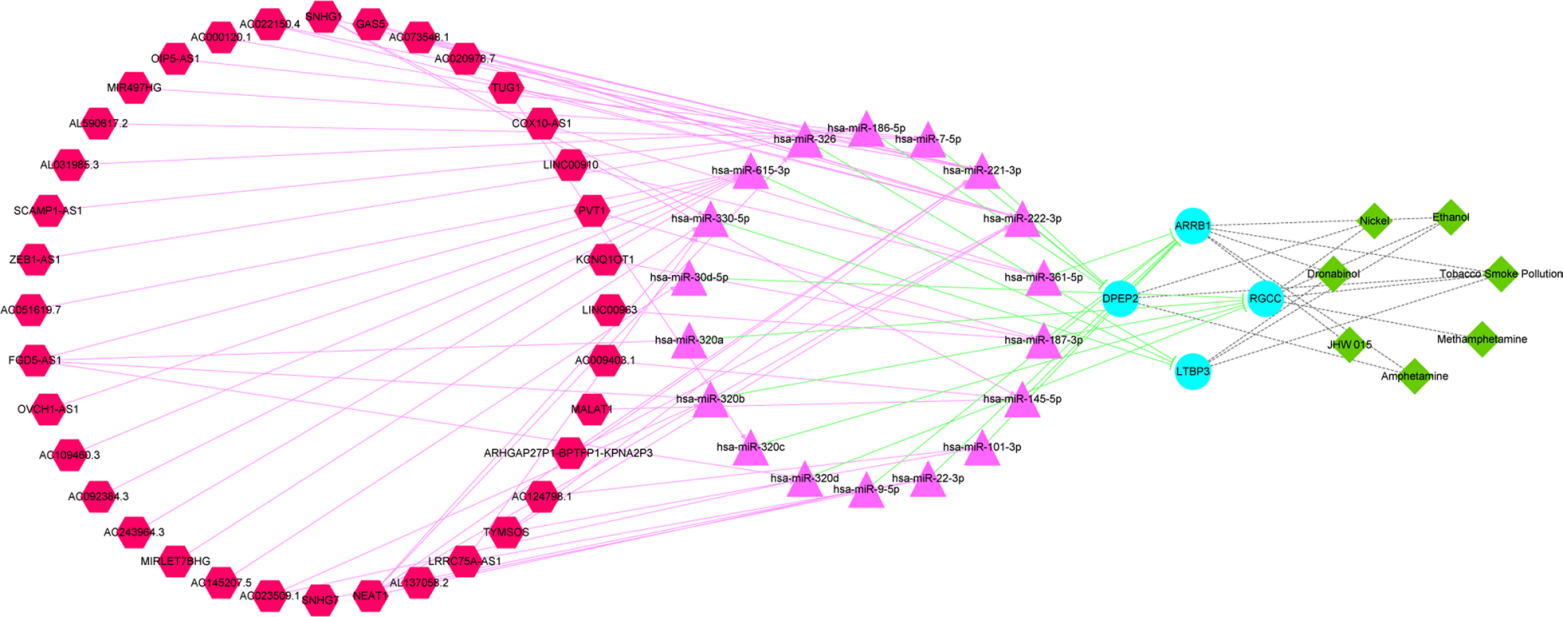
The lncRNA-miRNA-mRNA-drug regulatory network was constructed combining ceRNA network with potential therapeutic drugs (the red hexagon, purple triangle, light blue circle were represents lncRNA, miRNA and mRNA, seperately, and the green diamond represents molecular compound)

The ENCORI database was examined for miRNAs related to these four diagnostic genes. According to the ceRNA hypothesis, there is a negative association between miRNAs and lncRNAs or mRNAs. Therefore, the link between diagnostic markers (mRNA) and miRNAs was investigated, with miRNAs demonstrating a negative correlation with the diagnostic genes selected (Fig. 6A). Figure 6B shows the potential binding sites between miRNA sequences and diagnostic genes. Figure 6C illustrates the interaction of each diagnostic marker (mRNA) with targeted miRNAs. Furthermore, to construct the lncRNA-miRNA-mRNA regulatory network in HIV-1^+^, the ENCORI database was used to obtain the lncRNAs related to the above miRNAs, and the correlation between miRNAs and lncRNAs was calculated. Given multiple lncRNAs corresponding to the same miRNA, only the one that was most closely associated with miRNA was chosen for demonstration (Fig. 6D). Meanwhile, potential binding sites between lncRNA sequences and miRNAs were identified and documented in the ENCORI database (Fig. 6E). Based on 18 mRNA-miRNA pairs and 56 miRNA-lncRNA pairs, a ceRNA network in AIDS was constructed, as shown in Fig. 6F. Moreover, the lncRNA-miRNA-mRNA-drug regulatory network was developed using CTD-identified potential therapeutic medicines (Fig. 7). The red hexagon represents lncRNA, the purple triangle indicates miRNA, the light blue circle represents mRNA, and the green diamond indicates molecular compounds.

### ***Clinical sample validation of HIV-1***^+^***diagnostic genes***

**Fig. 8:**
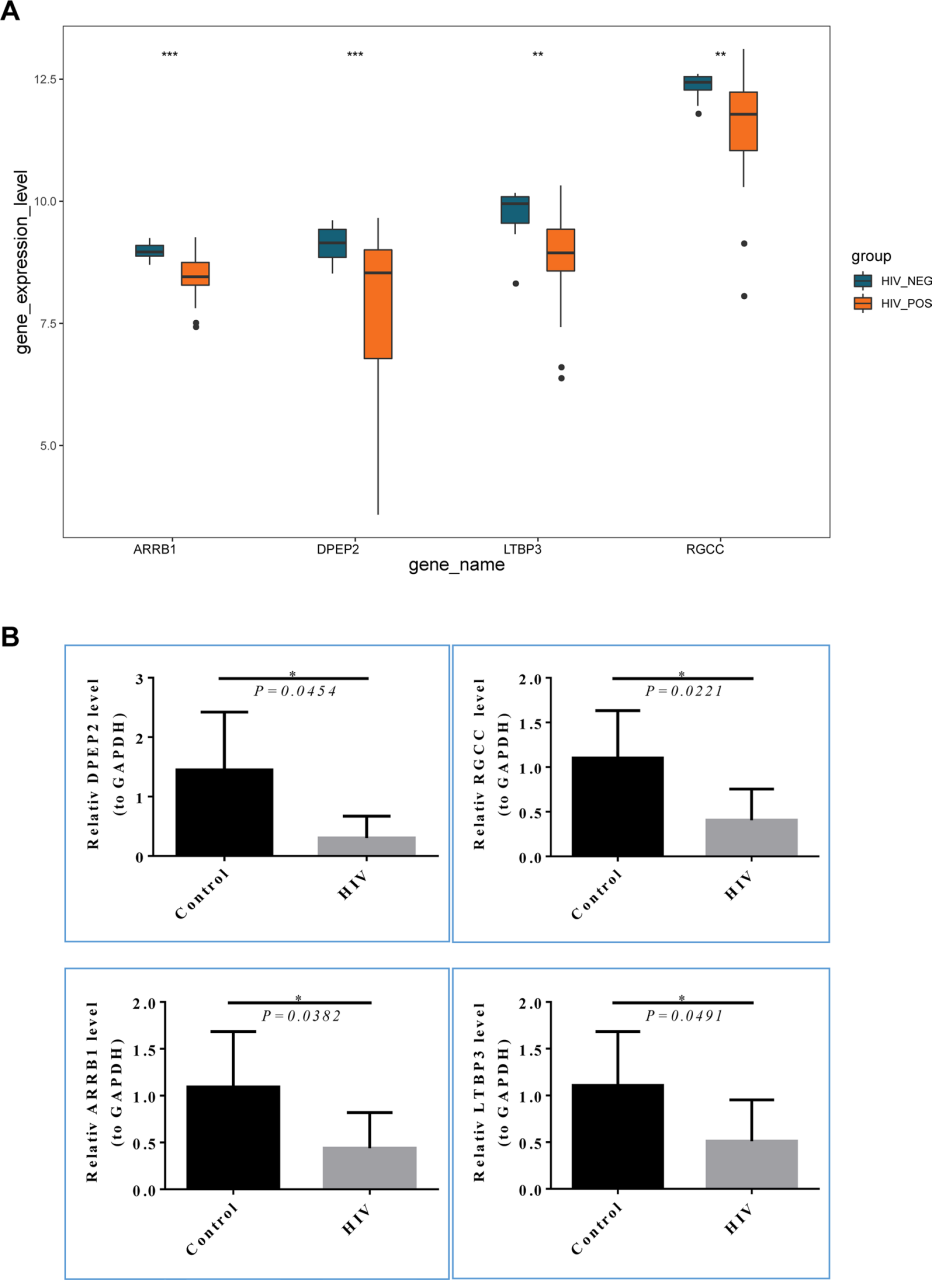
Clinical sample validation of HIV-1^+^ diagnostic genes. **A** the expression level of diagnostic genes in the GSE6740 dataset. **B** the relative expression of diagnostic genes was examined by RT-PCR in clinical blood samples

The expression levels of the four diagnostic genes were analyzed in the GSE6740 dataset, with the results suggesting that the expression levels of DPEP2, RGCC, ARRB1, and LTBP3 were significantly down-regulated (Fig. 8A). This is consistent with clinical blood sample detection results, which showed that the expression levels of four diagnostic genes were considerably down-regulated in the blood of HIV-1 + patients compared to the control group (Fig. 8B).

## Discussion

AIDS is a chronic disease characterized by a variety of life-threatening diseases resulting from HIV-1 infection. HIV-1 infection gradually weakens the immune system, increasing the risk of secondary infections and diseases [18]. Despite recent advancements in antiretroviral treatment to inhibit viral replication and contain the infection, treating AIDS remains a challenge [19, 20]. Therefore, it is critical to investigate the molecular markers and potential molecular pathways of HIV-1^+^ development for the early detection and treatment of HIV-1^+^.

The co-expression relationship between molecules was calculated using gene co-expression network analysis (WGCNA) in this study, and the results revealed that the MEdarkgreen module exhibited the most significant correlation with the clinical features of HIV-1, with 19 hub genes in the module identified. Among them, four immune cell-related genes (ARRB1, DPEP2, LTBP3, and RGCC) may show diagnostic value for HIV-1^+^.

Currently, there has been increasing evidence suggesting that these diagnostic genes related to immune cells are involved in the occurrence and development of various human diseases. As a member of the β-arrestins family, ARRB1 is essential for the survival of CD4^+^ T cells and has a role factor in autoimmune susceptibility [21, 22]. The most recent findings further indicate that ARRB1 can enhance the progression of primary biliary cirrhosis by regulating the function of autoimmune T cells [23]. DPEP2 was once believed to be a membrane-bound dipeptidase, but it was shown to be highly expressed in the lung, heart, and testis [24]. It has been shown that the expression of DPEP2 varies in inflammatory diseases, indicating that it may play a role in the immune response [25]. On the other hand, transforming growth factor-β binding protein 3 (LTBP3) plays an important role in the secretion, activation, and function of mature TGFβ [26]. Some studies are demonstrating that LTBP3 is also associated with a wide range of diseases, such as hepatocellular carcinoma, multiple myeloma, and oligodontia [27–29]. Additionally, by regulating angiogenesis-dependent intracellular perfusion, LTBP3 as a novel tumor target may promote early metastasis in the process of cancer cell proliferation [30]. RGCC participates in the regulation of the cell cycle through interaction with cyclin-dependent kinase 1 (CDK1) [31]. Moreover, RGCC has also been confirmed to be associated with inflammation, vascular remodeling, and insulin resistance.

To further understand the role these diagnostic genes play in HIV-1 infection, researchers investigated the molecular mechanisms in ARRB1, DPEP2, LTBP3, and RGCC. These genes, including ARRB1 and RGCC, were shown to be involved in the regulation of immune-related biological processes, which is consistent with the classical process required for HIV-1^+^ [32, 33], suggesting a potential mechanism of HIV-1 infection.

An increasing amount of research indicates that lncRNA-miRNA-mRNA regulatory networks play a role in disease pathogenesis and progression [34–36]. Herein, the ceRNA network of HIV-1^+^ was constructed to show that ARRB1, DPEP2, LTBP3, and RGCC can be regulated by lncRNAs through corresponding miRNAs, providing a foundation for further research into the complex regulatory mechanisms of HIV-1^+^. More importantly, we have also discovered the drugs that can be used to treat HIV-1^+^ and gained a better understanding of the molecular regulatory mechanisms of drug therapy for HIV-1^+^ through the lncRNA-miRNA-mRNA-drug regulatory network.

To the best of our knowledge, this is the first time ARRB1, DPEP2, LTBP3, and RGCC have been published as potential HIV-1^+^ diagnostic markers by WGCNA based on high-throughput sequencing data, alongside research into the molecular mechanisms underlying HIV-1^+^ development and treatment. In addition, the role of these four diagnostic genes in distinguishing HIV-1-infected and HIV-1-uninfected patients was further verified. Because many of the hub genes examined in WGCNA may be excluded, developing a diagnostic model with just the inherent limitations in consideration is inevitable. Besides, the potential diagnostic value of these genes was not verified by other external data sets.

## Conclusions

The ARRB1, DPEP2, LTBP3, and RGCC associations with immune cells may be identified as markers to distinguish HIV-1 infection using bioinformatics methods, and the potential mechanism of its molecular regulation was investigated, indicating a new direction for the early diagnosis and treatment of HIV-1^+^.

## Supplementary Information

**Additional file 1:** GSE6740 gene expression profile.

**Additional file 2**: **Table S1**. Differentially expressed genes in normal and HIV-1^+^ samples. **Table S2**. Results of GO analysis for 5 candidate genes. **Table S3**. Diagnostic genes and potential drugs.

## Data Availability

Publicly available datasets were analyzed in this study. This data can be found here: All the raw data used in this study are derived from the public GEO data portal (https://www.ncbi.nlm.nih.gov/geo/; Accession numbers: GSE6740). The datasets used and/or analysed during the current study are available from the corresponding author on reasonable request.
