# Supplementary figures and images for: Exploring the biological function of immune cell-related genes in human immunodeficiency virus (HIV)-1 infection based on weighted gene co-expression network analysis (WGCNA)
Source: BMC Med Genomics. 2022 Sep 19;15:200. doi: 10.1186/s12920-022-01357-y (PMC9484082; doi:10.1186/s12920-022-01357-y)

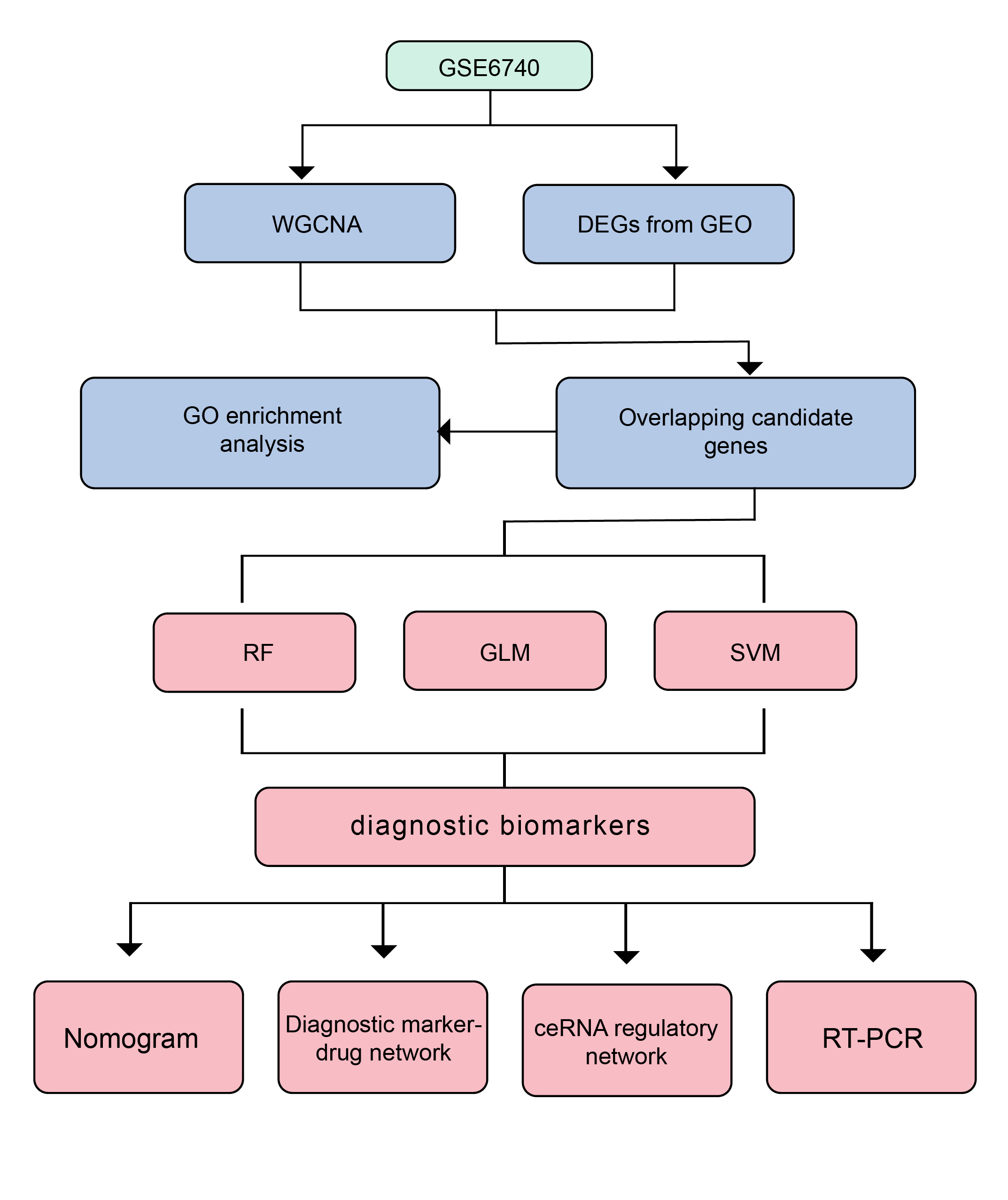

Supplement: Supplementary file 1 — Additional file 1: GSE6740 gene expression profile. [file 12920_2022_1357_MOESM1_ESM.jpg]
