# Supplementary material for: Exploring the biological function of immune cell-related genes in human immunodeficiency virus (HIV)-1 infection based on weighted gene co-expression network analysis (WGCNA)
Source: BMC Med Genomics. 2022 Sep 19;15:200. doi: 10.1186/s12920-022-01357-y (PMC9484082; doi:10.1186/s12920-022-01357-y)
Supplement: Supplementary file 2 — Additional file 2: Table S1. Differentially expressed genes in normal and HIV-1+ samples. Table S2. Results of GO analysis for 5 candidate genes. Table S3. Diagnostic genes and potential drugs. [file 12920_2022_1357_MOESM2_ESM.docx]

Supplementary Table 1. mRNA_diff.p.val0.05_lfc0.5

|  | logFC | AveExpr | t | P.Value | adj.P.Val | B |
| --- | --- | --- | --- | --- | --- | --- |
| IFI44L | 3.14801372 | 8.782297174 | 4.072523155 | 0.000209781 | 0.053978361 | 0.613013275 |
| IFI27 | 2.736225534 | 8.499404414 | 4.564933509 | 4.57E-05 | 0.036034382 | 1.95666539 |
| USP18 | 2.367492149 | 7.89007317 | 5.204998144 | 5.93E-06 | 0.013915487 | 3.756333124 |
| IFIT1 | 2.186541975 | 7.535490763 | 4.175709559 | 0.000153053 | 0.048670893 | 0.890497923 |
| CEP55 | 2.156178781 | 6.323740061 | 4.378312123 | 8.18E-05 | 0.040401792 | 1.441965907 |
| GINS2 | 2.117655696 | 5.483204908 | 3.114786 | 0.003370947 | 0.141989232 | -1.812083402 |
| ASPM | 2.078272588 | 4.792632538 | 3.669294942 | 0.000700203 | 0.074221565 | -0.444924816 |
| TOP2A | 1.847050036 | 6.855426534 | 4.197076141 | 0.000143336 | 0.04717696 | 0.948255734 |
| FAR2 | 1.821263025 | 6.331787213 | 4.837800624 | 1.92E-05 | 0.02651777 | 2.718547086 |
| IFI44 | 1.557936787 | 9.131770196 | 4.024160226 | 0.000242973 | 0.054788127 | 0.483822207 |
| CENPM | 1.543084691 | 6.614760768 | 3.878981983 | 0.000376245 | 0.064709277 | 0.099587758 |
| MELK | 1.517683775 | 7.339986599 | 4.293268466 | 0.000106552 | 0.045567678 | 1.209478914 |
| RRM2 | 1.474350575 | 8.503339131 | 3.379239567 | 0.001615577 | 0.101846663 | -1.174600929 |
| AIM2 | 1.465876123 | 8.480962217 | 5.950846001 | 5.26E-07 | 0.003261838 | 5.883113788 |
| DLGAP5 | 1.40786287 | 6.525037476 | 3.134329548 | 0.003195655 | 0.138092368 | -1.765973228 |
| MCM10 | 1.400883122 | 5.628509841 | 2.633277271 | 0.011908178 | 0.235763205 | -2.890881546 |
| MX1 | 1.396396979 | 10.49702688 | 3.473087978 | 0.001236565 | 0.092016384 | -0.941762595 |
| PCLAF | 1.389350549 | 9.039487606 | 4.062301602 | 0.000216406 | 0.053978361 | 0.58566125 |
| TYMS | 1.387274437 | 8.519333754 | 3.533640341 | 0.001038932 | 0.088133661 | -0.789836272 |
| OAS1 | 1.384917672 | 8.67812026 | 3.916228258 | 0.000336496 | 0.062905236 | 0.197633154 |
| CCR5 | 1.375328007 | 9.321380658 | 3.51557662 | 0.001094475 | 0.089756425 | -0.835293737 |
| NOD2 | 1.3600387 | 5.511423917 | 2.607771456 | 0.012692197 | 0.240318513 | -2.944695912 |
| CX3CR1 | 1.343307657 | 10.38376887 | 2.544141626 | 0.014858932 | 0.256270877 | -3.077361943 |
| H1-4 | 1.340144881 | 6.843454239 | 3.451553731 | 0.001315164 | 0.094383859 | -0.995476171 |
| ISG15 | 1.333383182 | 11.60435072 | 4.643834001 | 3.56E-05 | 0.036034382 | 2.175937298 |
| DHX58 | 1.308346051 | 6.408061058 | 3.16764128 | 0.002916567 | 0.130112474 | -1.686996969 |
| NUDT1 | 1.302070424 | 6.891851582 | 2.845045267 | 0.006926847 | 0.189639643 | -2.430612846 |
| CD38 | 1.294820581 | 8.457041932 | 3.623964498 | 0.000799421 | 0.078685912 | -0.560859404 |
| SHCBP1 | 1.284432391 | 6.094897406 | 3.078782949 | 0.003717878 | 0.147010235 | -1.896586763 |
| CDH12 | 1.281313833 | 4.479370929 | 2.335717928 | 0.024528303 | 0.315210144 | -3.495413204 |
| CD160 | 1.275532997 | 9.623028912 | 2.149583892 | 0.037598938 | 0.368020583 | -3.84608515 |
| APOL1 | 1.272032094 | 8.099883901 | 5.795118869 | 8.74E-07 | 0.003612232 | 5.438689501 |
| GEMIN6 | 1.258209609 | 5.914906748 | 3.324687593 | 0.00188443 | 0.108122832 | -1.30841539 |
| CHODL | 1.248176557 | 5.538107648 | 2.488217401 | 0.017036941 | 0.268137234 | -3.192055279 |
| BARD1 | 1.220309559 | 5.7128665 | 2.308347978 | 0.026150762 | 0.321747763 | -3.548358145 |
| MYBL2 | 1.218418469 | 6.92156281 | 2.79331667 | 0.007922878 | 0.199693849 | -2.545201356 |
| RSAD2 | 1.215516346 | 8.454670998 | 3.588129337 | 0.000887297 | 0.083365586 | -0.652032781 |
| GBP1 | 1.201467074 | 10.73772935 | 6.470345107 | 9.67E-08 | 0.001199854 | 7.359844604 |
| TPX2 | 1.20087941 | 7.722343637 | 4.194327765 | 0.000144551 | 0.04717696 | 0.940820794 |
| FBXL8 | 1.186330648 | 6.283764932 | 2.422681194 | 0.01995652 | 0.289136406 | -3.324134437 |
| TTK | 1.180835132 | 6.225242011 | 3.798741511 | 0.000477925 | 0.068281146 | -0.110316458 |
| SLC27A2 | 1.179016772 | 6.694343082 | 3.591469574 | 0.000878729 | 0.0831908 | -0.643552485 |
| GPR19 | 1.172415289 | 5.856411515 | 3.582627368 | 0.000901586 | 0.084071167 | -0.665993178 |
| DCLRE1A | 1.171823171 | 7.14182009 | 3.097135837 | 0.003537022 | 0.145145742 | -1.853582219 |
| LINC00574 | 1.160083221 | 5.949517844 | 2.665321056 | 0.010986344 | 0.228611806 | -2.822765903 |
| DDX60 | 1.158072676 | 10.3266423 | 5.001185293 | 1.14E-05 | 0.020226466 | 3.178886206 |
| PAGE4 | 1.149614997 | 6.185171629 | 2.61500746 | 0.012465087 | 0.239677537 | -2.929465364 |
| BRCA1 | 1.14865221 | 6.210506627 | 3.825036422 | 0.00044198 | 0.06737744 | -0.041733029 |
| HIVEP3 | 1.143849946 | 6.70932726 | 3.941354726 | 0.000312016 | 0.062905236 | 0.263986549 |
| IGHD | 1.138533745 | 7.243333435 | 2.692930736 | 0.010245289 | 0.223546489 | -2.763628557 |
| IFI6 | 1.135133121 | 9.781650567 | 4.387319796 | 7.96E-05 | 0.040401792 | 1.466670421 |
| SPATS2L | 1.121122179 | 8.475908558 | 3.719166996 | 0.00060476 | 0.071591261 | -0.316618375 |
| SPC25 | 1.113215475 | 6.228033509 | 2.985649108 | 0.004777865 | 0.161603064 | -2.112465208 |
| NUSAP1 | 1.109402779 | 8.118149807 | 3.841321239 | 0.000421046 | 0.066098921 | 0.000841516 |
| KIF15 | 1.101043041 | 6.448762199 | 3.074830437 | 0.003757952 | 0.147021894 | -1.905828617 |
| NCAPG | 1.094027689 | 6.409055151 | 3.072948556 | 0.003777174 | 0.147021894 | -1.910226415 |
| JAM2 | 1.078192292 | 6.925683188 | 2.578726148 | 0.013642544 | 0.247533015 | -3.005537351 |
| ZNF80 | 1.054793534 | 4.659912687 | 2.977261083 | 0.004886129 | 0.162124851 | -2.131711268 |
| RTP4 | 1.051366381 | 7.752743098 | 4.343427565 | 9.12E-05 | 0.040401792 | 1.346433438 |
| HLA-DRA | 1.043842651 | 10.08795802 | 2.324771647 | 0.025166013 | 0.317517644 | -3.516643813 |
| IFNA8 | 1.042107977 | 3.593024101 | 2.272097706 | 0.028447487 | 0.332919268 | -3.617759343 |
| LAMP3 | 1.040948528 | 8.206845654 | 2.409649169 | 0.020588485 | 0.292819253 | -3.350094029 |
| XAF1 | 1.035504034 | 10.04233398 | 3.701741773 | 0.000636586 | 0.071591261 | -0.36153703 |
| TMEM156 | 1.033880052 | 6.882362884 | 3.931136189 | 0.000321756 | 0.062905236 | 0.23698136 |
| KIF20A | 1.029913583 | 5.000294732 | 2.130755953 | 0.039215098 | 0.372954439 | -3.880304451 |
| KIF11 | 1.026540078 | 7.012629787 | 2.967300026 | 0.005017685 | 0.162478656 | -2.154523571 |
| PLSCR1 | 1.024883308 | 9.481356047 | 3.818041142 | 0.000451279 | 0.06737744 | -0.059997939 |
| IFIT3 | 1.015657912 | 8.729391852 | 3.406570485 | 0.001495049 | 0.099685985 | -1.107130818 |
| RAG1 | 1.015483304 | 5.157316573 | 2.137216779 | 0.038653743 | 0.371009363 | -3.868588586 |
| LAG3 | 1.007525301 | 8.992848597 | 2.575991004 | 0.013735315 | 0.247533015 | -3.011242301 |
| ZNF234 | 0.998054907 | 5.336063187 | 2.576059922 | 0.01373297 | 0.247533015 | -3.011098604 |
| GIMAP4 | 0.995441195 | 10.94344798 | 4.42078288 | 7.17E-05 | 0.040401792 | 1.558575375 |
| RPS2P45 | 0.991899445 | 5.679261011 | 3.182127075 | 0.002802543 | 0.128293771 | -1.652505208 |
| CDCA3 | 0.988208976 | 6.214010731 | 2.434998114 | 0.019375429 | 0.285551723 | -3.299505783 |
| FOXM1 | 0.988119183 | 5.344640378 | 2.41383523 | 0.020383543 | 0.291240441 | -3.341766584 |
| ESM1 | 0.981519602 | 5.478310814 | 3.085374228 | 0.003651944 | 0.145631557 | -1.881159333 |
| HERC6 | 0.972104314 | 9.242743582 | 3.90162896 | 0.000351566 | 0.063190101 | 0.159157554 |
| CPA1 | 0.965448072 | 3.414342997 | 3.091233276 | 0.003594263 | 0.145145742 | -1.867429477 |
| CDC42EP1 | 0.96537073 | 6.417376533 | 2.414115968 | 0.020369865 | 0.291240441 | -3.341207726 |
| IRF7 | 0.961025897 | 10.32632207 | 3.995640414 | 0.000264884 | 0.057633086 | 0.407908736 |
| BRDT | 0.950876195 | 3.8484875 | 2.329980241 | 0.024860737 | 0.316228573 | -3.506550875 |
| FANCL | 0.926702521 | 6.86662694 | 5.455204814 | 2.64E-06 | 0.008180873 | 4.468527715 |
| DTL | 0.9239293 | 7.301759683 | 2.740046126 | 0.009086382 | 0.213922091 | -2.661770208 |
| H2AC4 | 0.919813005 | 6.541702901 | 2.912077257 | 0.005809273 | 0.174250824 | -2.28013438 |
| AKAP4 | 0.91497835 | 5.407490092 | 2.464292601 | 0.018054485 | 0.277260134 | -3.240566518 |
| CDC20 | 0.911617632 | 6.227435154 | 2.23674687 | 0.030859093 | 0.341392724 | -3.684636604 |
| FANCI | 0.905897428 | 7.04394445 | 3.411505387 | 0.001474222 | 0.098828644 | -1.094918376 |
| SLC13A1 | 0.905274225 | 2.83084702 | 2.546671058 | 0.014766716 | 0.255420933 | -3.07213189 |
| AURKB | 0.90154717 | 6.801204188 | 2.200601968 | 0.033511436 | 0.354750958 | -3.752186738 |
| DMRT1 | 0.901357439 | 5.826191384 | 2.513519484 | 0.016017837 | 0.262224328 | -3.140387778 |
| ERC2-IT1 | 0.901317536 | 6.055550509 | 2.264107572 | 0.028977346 | 0.335866392 | -3.632944835 |
| NFIX | 0.900810978 | 5.662623684 | 2.45266324 | 0.018568767 | 0.280157965 | -3.264025175 |
| AURKA | 0.897729522 | 6.377002483 | 3.324310688 | 0.001886428 | 0.108122832 | -1.309335903 |
| TYMP | 0.896628097 | 8.032012975 | 4.158684399 | 0.000161253 | 0.049996602 | 0.844547651 |
| ECT2 | 0.896387188 | 6.310593985 | 3.735057542 | 0.000577079 | 0.070860788 | -0.275574061 |
| RAD51 | 0.896085305 | 5.6212419 | 2.11466071 | 0.040644876 | 0.378160103 | -3.909370094 |
| PCBD1 | 0.894658553 | 6.345805104 | 2.898557673 | 0.006020128 | 0.176923287 | -2.310662087 |
| IFI35 | 0.893624289 | 9.724869271 | 4.032042273 | 0.000237235 | 0.054788127 | 0.504838314 |
| RAD51AP1 | 0.892403446 | 6.20866191 | 2.242389452 | 0.030462343 | 0.340507917 | -3.674015412 |
| CCNO | 0.886935419 | 6.032897318 | 2.521231812 | 0.015718441 | 0.26083155 | -3.12456524 |
| EDN3 | 0.885896608 | 4.980189684 | 2.991376447 | 0.00470524 | 0.160755888 | -2.099305052 |
| MED18 | 0.869253419 | 4.988604818 | 3.111861423 | 0.003397946 | 0.141989232 | -1.818969122 |
| HSD3B1 | 0.868776024 | 3.956123507 | 2.284823244 | 0.027621462 | 0.330657697 | -3.593490583 |
| PBK | 0.866856858 | 6.509691576 | 3.175551908 | 0.002853766 | 0.129570038 | -1.668172247 |
| RPE | 0.858002581 | 6.971485288 | 2.999157028 | 0.004608238 | 0.159949701 | -2.08140244 |
| CDR1 | 0.855678646 | 3.070050406 | 2.521666285 | 0.015701728 | 0.26083155 | -3.123672859 |
| LPA | 0.845666074 | 4.290684286 | 2.152501708 | 0.037353808 | 0.366794874 | -3.840761067 |
| CCNA2 | 0.845187939 | 7.601594898 | 3.191369892 | 0.002732008 | 0.126900259 | -1.630450802 |
| ANOS1 | 0.844657798 | 6.597253418 | 2.567964318 | 0.014010912 | 0.249666562 | -3.027959964 |
| POU2AF1 | 0.836858151 | 8.033608324 | 2.971289943 | 0.004964596 | 0.162456256 | -2.145391689 |
| GINS1 | 0.836852735 | 6.648907661 | 2.202782267 | 0.033345887 | 0.354750958 | -3.748136002 |
| STAT1 | 0.834877022 | 11.29332887 | 5.165516747 | 6.73E-06 | 0.013915487 | 3.64424455 |
| LMNB1 | 0.833783903 | 8.693656516 | 3.091335052 | 0.003593268 | 0.145145742 | -1.867190846 |
| CDK1 | 0.830522199 | 7.228664194 | 3.090171509 | 0.003604652 | 0.145145742 | -1.869918713 |
| OAS3 | 0.829572496 | 9.483279641 | 3.506545284 | 0.001123302 | 0.090315937 | -0.857978253 |
| CYP4F3 | 0.829124638 | 5.643490289 | 2.662832066 | 0.011055521 | 0.228899115 | -2.828076825 |
| GPM6A | 0.8283244 | 4.280486767 | 3.147512868 | 0.003082313 | 0.134601568 | -1.734775149 |
| MSX1 | 0.826493751 | 6.094467947 | 2.352463678 | 0.023580802 | 0.30946995 | -3.462790906 |
| NDUFAF7 | 0.825396628 | 5.052791636 | 2.16761583 | 0.0361065 | 0.360832242 | -3.813092693 |
| KIF1C | 0.821295123 | 5.238213344 | 2.023326855 | 0.049657151 | 0.409556494 | -4.070994228 |
| CXCL11 | 0.819196387 | 4.28700122 | 2.398275186 | 0.021154776 | 0.295027399 | -3.37266727 |
| TK1 | 0.8147362 | 7.581660573 | 2.398065623 | 0.02116534 | 0.295027399 | -3.373082443 |
| IFNG | 0.807252132 | 9.03083972 | 2.321869868 | 0.025337543 | 0.317721823 | -3.522259413 |
| TNFSF10 | 0.806705327 | 9.095040769 | 2.629562332 | 0.012019531 | 0.235864279 | -2.89874201 |
| NCAPH | 0.806291258 | 5.467818989 | 2.104653559 | 0.041556764 | 0.378729629 | -3.927354173 |
| MT1E | 0.799578648 | 10.32729224 | 3.629214924 | 0.000787271 | 0.078109913 | -0.547465391 |
| ZBP1 | 0.798777582 | 7.384922075 | 3.182017758 | 0.002803388 | 0.128293771 | -1.652765835 |
| FABP5 | 0.798136463 | 9.518311124 | 2.239524317 | 0.03066323 | 0.341313226 | -3.679411088 |
| DRP2 | 0.797930481 | 5.846401592 | 2.788498752 | 0.008022123 | 0.199693849 | -2.555804543 |
| DEPDC1 | 0.797802332 | 6.567093669 | 2.79857597 | 0.007815843 | 0.199651163 | -2.533613187 |
| OAS2 | 0.786675394 | 9.321176818 | 3.712137988 | 0.000617409 | 0.071591261 | -0.334749059 |
| LGALS9 | 0.776906531 | 8.469149273 | 2.595787386 | 0.013076698 | 0.243125289 | -2.969856224 |
| IFIH1 | 0.774885778 | 7.602379603 | 4.132013299 | 0.000174968 | 0.051996414 | 0.77269382 |
| GPR171 | 0.774186388 | 9.969038032 | 3.964436383 | 0.000291057 | 0.062235923 | 0.325087215 |
| NCAPG2 | 0.770128567 | 8.200517493 | 2.896123532 | 0.006058843 | 0.177221153 | -2.316148992 |
| FDXR | 0.769994835 | 6.802970707 | 3.067214625 | 0.003836317 | 0.147021894 | -1.923616321 |
| KRT24 | 0.769694608 | 5.948175517 | 2.046403093 | 0.047229492 | 0.402149969 | -4.030695094 |
| PLK4 | 0.765533559 | 5.503795683 | 2.734914648 | 0.009206443 | 0.214218213 | -2.672920943 |
| GNRH1 | 0.764300238 | 4.821425373 | 2.473722977 | 0.017646985 | 0.274947125 | -3.221485081 |
| CHI3L2 | 0.763759064 | 9.132895535 | 3.052598863 | 0.003991043 | 0.148337431 | -1.957680073 |
| HAS1 | 0.763499174 | 5.430181203 | 2.217204753 | 0.032268958 | 0.348605934 | -3.721263088 |
| CCNF | 0.762864971 | 6.501313356 | 2.854986364 | 0.006749321 | 0.187321692 | -2.408436853 |
| EZH2 | 0.762082641 | 8.306731643 | 4.059349834 | 0.000218357 | 0.053978361 | 0.577767243 |
| RAB17 | 0.758797602 | 6.179854374 | 2.657377828 | 0.011208517 | 0.229805623 | -2.839703119 |
| TESC | 0.758248073 | 8.431141204 | 2.441820968 | 0.019060178 | 0.283395462 | -3.285823987 |
| TMEM254 | 0.75536641 | 6.613999199 | 3.127478664 | 0.003256098 | 0.13877021 | -1.782155896 |
| HCG18 | 0.753262558 | 5.408724896 | 2.246225799 | 0.030195197 | 0.339511186 | -3.666782498 |
| THRAP3 | 0.749038254 | 6.463970897 | 2.03626652 | 0.048282833 | 0.40521307 | -4.048442132 |
| ZWILCH | 0.748937573 | 7.637360605 | 3.553173596 | 0.000981916 | 0.086366788 | -0.740553193 |
| GLMN | 0.748130257 | 6.940623412 | 2.496028477 | 0.016716203 | 0.265107859 | -3.176144578 |
| C19orf57 | 0.744249428 | 6.509517852 | 2.456578076 | 0.018394176 | 0.279222247 | -3.256137122 |
| ARMT1 | 0.740902325 | 8.209069552 | 3.916578995 | 0.000336142 | 0.062905236 | 0.19855821 |
| DGCR11 | 0.738133178 | 7.40075198 | 2.676051101 | 0.010692664 | 0.22746212 | -2.799832041 |
| COG7 | 0.737023199 | 7.108592495 | 2.530163395 | 0.015378098 | 0.259574319 | -3.10619846 |
| TAP1 | 0.736540831 | 10.88288692 | 3.390615292 | 0.001564325 | 0.100790971 | -1.146552683 |
| CLDND1 | 0.732838468 | 9.347636142 | 3.648695411 | 0.000743725 | 0.077132477 | -0.497691421 |
| HERC5 | 0.731704473 | 9.223987813 | 2.878754641 | 0.006341917 | 0.180810237 | -2.355216613 |
| NPR2 | 0.722875347 | 5.933652577 | 2.10848699 | 0.041205346 | 0.378160103 | -3.920472961 |
| SCO2 | 0.719861169 | 8.785862021 | 4.044603953 | 0.000228362 | 0.054464243 | 0.538363523 |
| HJURP | 0.719411486 | 6.700382455 | 2.066367046 | 0.045213431 | 0.394628173 | -3.995536882 |
| NRIP2 | 0.712816514 | 4.987422126 | 2.231483251 | 0.031233338 | 0.34309642 | -3.694526042 |
| SMC2 | 0.711781053 | 7.352616579 | 3.798809349 | 0.000477829 | 0.068281146 | -0.110139779 |
| TMEM187 | 0.710826566 | 7.562749098 | 2.981903799 | 0.004825925 | 0.161759775 | -2.121062786 |
| PTPRN2 | 0.700051922 | 6.111064114 | 2.450819689 | 0.018651504 | 0.281064336 | -3.267736627 |
| BUB1 | 0.698731411 | 5.444736051 | 3.397803218 | 0.001532744 | 0.10040969 | -1.128804719 |
| OASL | 0.688742702 | 9.520474321 | 3.769891914 | 0.000520613 | 0.06839991 | -0.18533026 |
| MCM4 | 0.685788283 | 7.375400027 | 2.630111733 | 0.012003003 | 0.235864279 | -2.897580008 |
| KLHL5 | 0.685480613 | 6.174271764 | 2.529120753 | 0.015417479 | 0.259574319 | -3.108344899 |
| NEK2 | 0.680742832 | 6.306431859 | 2.880320864 | 0.006315893 | 0.180483199 | -2.351699815 |
| KIF14 | 0.680692118 | 6.526333437 | 2.611969906 | 0.012559966 | 0.240304388 | -2.935862434 |
| CCDC28B | 0.67867054 | 6.585688461 | 2.821468642 | 0.007365408 | 0.194352752 | -2.483007716 |
| KLF6 | 0.677733112 | 10.91480409 | 3.017919383 | 0.004381993 | 0.154830426 | -2.038115727 |
| ECE2 | 0.676896383 | 6.974652379 | 2.088370458 | 0.04307896 | 0.386588464 | -3.956473048 |
| OIP5 | 0.67384703 | 6.959616728 | 3.071727623 | 0.003789695 | 0.147021894 | -1.913078782 |
| PTTG3P | 0.672031014 | 6.412240453 | 2.130652743 | 0.039224123 | 0.372954439 | -3.880491385 |
| MYO1F | 0.670514748 | 10.95833099 | 2.457624966 | 0.018347741 | 0.279200844 | -3.254026194 |
| ABCA6 | 0.668231655 | 4.822660601 | 2.179136735 | 0.035180603 | 0.359181911 | -3.791901253 |
| TMEM143 | 0.667298223 | 5.684336243 | 2.05519383 | 0.04633229 | 0.39876975 | -4.015247378 |
| WIPI1 | 0.666850402 | 6.970545861 | 2.372182931 | 0.022507374 | 0.304070203 | -3.424154801 |
| CRH | 0.66631219 | 4.977986217 | 2.072574759 | 0.04460207 | 0.392030387 | -3.984549241 |
| FBXO5 | 0.662380409 | 7.537580916 | 2.75310385 | 0.008787378 | 0.210388145 | -2.633333356 |
| FGF2 | 0.660617949 | 4.738111407 | 2.123919649 | 0.039816882 | 0.376378791 | -3.892670936 |
| KIF4A | 0.659305086 | 6.692390641 | 2.22601011 | 0.031626758 | 0.344973658 | -3.704790242 |
| EIF2AK2 | 0.658408009 | 9.83028099 | 3.513808343 | 0.001100063 | 0.089756425 | -0.839737479 |
| IGLL3P | 0.657322295 | 8.592122889 | 2.357409579 | 0.023307325 | 0.308142575 | -3.453122769 |
| RBBP8 | 0.656260101 | 8.445422297 | 3.026355918 | 0.004283701 | 0.152899232 | -2.018598727 |
| SAMD9 | 0.654509407 | 8.208275655 | 2.886527401 | 0.006213745 | 0.178386276 | -2.337751773 |
| APOBEC3B | 0.653718716 | 6.793805002 | 2.65674934 | 0.011226271 | 0.229805623 | -2.841041767 |
| PRDX3 | 0.652747766 | 8.862083071 | 3.211520226 | 0.002584022 | 0.123284731 | -1.582245046 |
| HMMR | 0.650961728 | 7.272128053 | 2.608407755 | 0.012672075 | 0.240304388 | -2.943357777 |
| CHEK1 | 0.650758861 | 6.707803038 | 2.327170538 | 0.025024998 | 0.317341544 | -3.511997462 |
| PSMA5 | 0.650033681 | 10.6472197 | 3.61520675 | 0.00082009 | 0.079938057 | -0.583180551 |
| IRX5 | 0.648217735 | 5.926235708 | 2.155989384 | 0.037062655 | 0.364513125 | -3.834389791 |
| C1GALT1C1 | 0.643333816 | 7.847135466 | 2.976661199 | 0.004893959 | 0.162124851 | -2.133086414 |
| TTF2 | 0.642023349 | 7.263993654 | 2.558329783 | 0.014348394 | 0.253007896 | -3.047978272 |
| ZWINT | 0.641234023 | 9.040517465 | 2.641795456 | 0.011656423 | 0.23429976 | -2.872829218 |
| ACOT7 | 0.638790021 | 7.884434587 | 3.357183195 | 0.001719551 | 0.101846663 | -1.228842998 |
| LIM2 | 0.637471607 | 6.841773013 | 2.176635321 | 0.035379828 | 0.359181911 | -3.796509729 |
| INTS7 | 0.636343869 | 6.920984427 | 3.047811826 | 0.004042984 | 0.149229431 | -1.968815801 |
| LYVE1 | 0.63125988 | 5.550233579 | 2.235595571 | 0.030940608 | 0.341392724 | -3.686801217 |
| S100A11 | 0.630294036 | 9.525972207 | 3.27813524 | 0.00214706 | 0.113259951 | -1.421684491 |
| C1R | 0.6299013 | 6.757539641 | 2.337112444 | 0.024448113 | 0.315104897 | -3.492703167 |
| ATP1A2 | 0.629212168 | 5.784483728 | 2.254722695 | 0.029610932 | 0.337842488 | -3.650729253 |
| FAS | 0.627706088 | 9.417637466 | 4.378271786 | 8.19E-05 | 0.040401792 | 1.441855313 |
| GCNT1 | 0.620655495 | 6.77018597 | 2.265119479 | 0.028909758 | 0.335685991 | -3.631023917 |
| CHST12 | 0.615899992 | 9.694814173 | 2.0434439 | 0.047534905 | 0.403299672 | -4.035883308 |
| JRKL | 0.615878919 | 7.796687283 | 2.546723537 | 0.014764808 | 0.255420933 | -3.072023342 |
| SH2D1A | 0.614920691 | 10.0260721 | 2.658720593 | 0.011170671 | 0.229805623 | -2.836842374 |
| GALK1 | 0.613134724 | 4.563000348 | 2.107016652 | 0.041339825 | 0.378160103 | -3.923113461 |
| NSUN7 | 0.613064942 | 6.343198551 | 2.405963595 | 0.020770464 | 0.294731458 | -3.357417107 |
| KRT3 | 0.613027111 | 6.582598272 | 2.691357807 | 0.010286236 | 0.223546489 | -2.767008655 |
| UNG | 0.61264117 | 8.404441346 | 3.434255279 | 0.001381743 | 0.096271786 | -1.038501787 |
| TIPRL | 0.611772782 | 8.628726426 | 3.758196965 | 0.000538948 | 0.06839991 | -0.215668625 |
| CALML4 | 0.610301339 | 5.22564643 | 2.174223019 | 0.035572901 | 0.359672489 | -3.800950145 |
| MIS18BP1 | 0.61003147 | 6.996684864 | 2.434637135 | 0.019392239 | 0.285551723 | -3.300228878 |
| BATF | 0.609926688 | 9.740108743 | 3.054859805 | 0.00396673 | 0.148337431 | -1.952416999 |
| NUP43 | 0.603937156 | 8.208518772 | 2.201154177 | 0.033469438 | 0.354750958 | -3.751161091 |
| LXN | 0.595655061 | 8.103652151 | 2.524927821 | 0.015576778 | 0.260693194 | -3.116970387 |
| CCDC71 | 0.594032582 | 6.501415132 | 2.170425091 | 0.035878764 | 0.35972485 | -3.807933397 |
| EDN2 | 0.591328335 | 4.737512712 | 2.177132266 | 0.03534017 | 0.359181911 | -3.795594508 |
| DCPS | 0.587280279 | 8.99544437 | 3.163137107 | 0.002952907 | 0.130792702 | -1.69770349 |
| TCF15 | 0.585559928 | 3.974196918 | 2.984295026 | 0.004795188 | 0.161603064 | -2.115574342 |
| IFI16 | 0.584475583 | 11.22438002 | 4.380369239 | 8.13E-05 | 0.040401792 | 1.447606457 |
| ELAC2 | 0.582169647 | 7.621932191 | 3.927908796 | 0.000324893 | 0.062905236 | 0.22845789 |
| RACGAP1 | 0.580528815 | 8.341853345 | 3.280257242 | 0.002134367 | 0.11312146 | -1.416540153 |
| PAAF1 | 0.58040036 | 7.680490108 | 2.142250202 | 0.038221329 | 0.369419849 | -3.859441911 |
| RHBG | 0.580003704 | 4.971834898 | 2.985305325 | 0.004782258 | 0.161603064 | -2.113254656 |
| WDFY3 | 0.577943172 | 4.976708122 | 2.037966814 | 0.048104731 | 0.405024338 | -4.045470189 |
| HCP5 | 0.577672099 | 8.447938663 | 2.468085426 | 0.017889575 | 0.277240286 | -3.232898402 |
| MYL6B | 0.576628005 | 8.134729974 | 2.672386801 | 0.010792131 | 0.227754622 | -2.807670941 |
| MRPL19 | 0.573317246 | 7.605959628 | 4.010622464 | 0.000253144 | 0.0560624 | 0.447762194 |
| PTTG1 | 0.573284487 | 10.2730608 | 3.036530647 | 0.004167916 | 0.15203087 | -1.995017113 |
| GPR15 | 0.569869866 | 7.973886122 | 2.278527825 | 0.028027384 | 0.332218692 | -3.605509318 |
| PSMA4 | 0.567670272 | 10.9348867 | 3.509722073 | 0.00111308 | 0.090224945 | -0.850002209 |
| MKI67 | 0.566240049 | 7.60098474 | 2.670659708 | 0.010839309 | 0.227754622 | -2.811363128 |
| BST2 | 0.562912011 | 10.05408041 | 3.672761345 | 0.000693125 | 0.074221565 | -0.436032118 |
| PDPN | 0.562524906 | 5.042977944 | 2.207782074 | 0.032968991 | 0.35330217 | -3.738835301 |
| CASP1 | 0.560497578 | 10.4020472 | 4.906554916 | 1.55E-05 | 0.023957254 | 2.911939913 |
| IRS4 | 0.554134568 | 7.495302649 | 2.207919628 | 0.032958676 | 0.35330217 | -3.738579193 |
| NPTX1 | 0.552270574 | 6.843646193 | 2.073332367 | 0.044527956 | 0.391934496 | -3.983206484 |
| GMNN | 0.551696405 | 8.603760233 | 2.691069073 | 0.010293769 | 0.223546489 | -2.767628975 |
| CASP7 | 0.551589827 | 9.174614172 | 4.66240194 | 3.36E-05 | 0.036034382 | 2.227669295 |
| TRIM22 | 0.549289331 | 12.03648793 | 3.42087184 | 0.001435452 | 0.09781583 | -1.07171414 |
| MT1HL1 | 0.547460971 | 11.28024275 | 2.209646054 | 0.032829451 | 0.353052832 | -3.735363756 |
| MRE11 | 0.547233748 | 7.1237414 | 2.785315179 | 0.008088335 | 0.199693849 | -2.562804343 |
| CPTP | 0.54489803 | 8.249065692 | 2.029882716 | 0.048956659 | 0.406944022 | -4.059582646 |
| HRH2 | 0.543590847 | 6.574785896 | 2.156060185 | 0.037056766 | 0.364513125 | -3.834260369 |
| ETNK1 | 0.543537398 | 7.554546087 | 2.750610744 | 0.008843753 | 0.210892838 | -2.638769688 |
| CISD1 | 0.542941791 | 8.853669126 | 3.893033104 | 0.000360742 | 0.063913201 | 0.136530764 |
| TMPRSS3 | 0.542424351 | 7.212238269 | 3.857034102 | 0.000401759 | 0.064709277 | 0.041992177 |
| PEX13 | 0.541910135 | 7.18846819 | 2.621183982 | 0.012274189 | 0.238718485 | -2.916441873 |
| TEFM | 0.541364396 | 7.619392468 | 3.316408624 | 0.001928783 | 0.109227256 | -1.32862224 |
| SCG5 | 0.537945382 | 6.304216779 | 3.482620423 | 0.001203227 | 0.091548562 | -0.917932026 |
| SRRD | 0.537697155 | 8.286661306 | 3.069717429 | 0.003810396 | 0.147021894 | -1.917773578 |
| MAGOH2P | 0.534792814 | 6.817047965 | 2.441693706 | 0.019066015 | 0.283395462 | -3.286079438 |
| EEF1AKNMT | 0.532093071 | 8.197150483 | 3.464933072 | 0.001265786 | 0.092806307 | -0.962123417 |
| PSMB10 | 0.525400863 | 11.04535669 | 3.066888223 | 0.00383971 | 0.147021894 | -1.924378091 |
| KIF18B | 0.524657861 | 7.224284646 | 2.117063197 | 0.040428588 | 0.378160103 | -3.905042543 |
| DNA2 | 0.523847051 | 6.776397386 | 2.722344301 | 0.009506781 | 0.217913378 | -2.700177731 |
| COL1A2 | 0.523534351 | 6.633931141 | 2.646155283 | 0.011529471 | 0.233260187 | -2.863574167 |
| SMC4 | 0.522339225 | 9.125848385 | 3.484990049 | 0.001195074 | 0.091489527 | -0.912003037 |
| SEM1 | 0.520587938 | 10.17085803 | 2.815074819 | 0.00748872 | 0.195939051 | -2.497168442 |
| IL10 | 0.519734605 | 6.66325058 | 2.310457814 | 0.026022353 | 0.320978269 | -3.544293444 |
| CLSTN3 | 0.518390423 | 7.462833535 | 2.137956174 | 0.038589953 | 0.371002018 | -3.867246021 |
| FRMD8 | 0.513993206 | 5.338586283 | 2.05369514 | 0.046484189 | 0.398963223 | -4.017884718 |
| HMGB3 | 0.513411077 | 7.847926053 | 3.275428209 | 0.002163357 | 0.113259951 | -1.428244473 |
| CTSC | 0.512828641 | 10.4674453 | 2.198582215 | 0.033665446 | 0.355335196 | -3.755936441 |
| USP46 | 0.512216441 | 7.042893094 | 2.785544827 | 0.008083542 | 0.199693849 | -2.562299585 |
| SIT1 | 0.511788363 | 10.06784953 | 2.358699172 | 0.023236489 | 0.308142575 | -3.450599431 |
| ASB4 | 0.511696439 | 5.26868325 | 2.033623603 | 0.048560813 | 0.405283446 | -4.053057741 |
| MICB | 0.510675096 | 9.88773572 | 2.906117304 | 0.005901361 | 0.174258756 | -2.293603144 |
| MTHFD2 | 0.510345034 | 10.08115116 | 3.817590216 | 0.000451885 | 0.06737744 | -0.061174836 |
| EMC9 | 0.510137151 | 7.274721779 | 2.08287089 | 0.043604007 | 0.389048124 | -3.966267462 |
| HSPB11 | 0.509400205 | 9.38746932 | 3.59707932 | 0.000864518 | 0.082474985 | -0.629301878 |
| E2F1 | 0.508525085 | 6.948920519 | 3.412449911 | 0.001470267 | 0.098828644 | -1.092579917 |
| WDHD1 | 0.501680199 | 5.651140447 | 2.168441081 | 0.036039468 | 0.360452807 | -3.811577632 |
| JPT1 | 0.501367861 | 9.503087399 | 3.201410987 | 0.002657289 | 0.12578511 | -1.606450749 |
| DENND2D | 0.500110692 | 11.07366427 | 2.454276566 | 0.018496636 | 0.279666262 | -3.260775562 |
| GOLGA8N | -0.500025872 | 9.917067416 | -2.672231746 | 0.010796359 | 0.227754622 | -2.808002485 |
| RBM38 | -0.500313545 | 11.1509293 | -2.550478598 | 0.014628889 | 0.255420933 | -3.064252241 |
| PTP4A1 | -0.501019495 | 11.00804317 | -3.259421332 | 0.00226215 | 0.114510943 | -1.466973564 |
| NLE1 | -0.501627606 | 7.646012868 | -4.719914527 | 2.80E-05 | 0.034710491 | 2.388201003 |
| HSF2 | -0.501930346 | 8.176308944 | -3.176831932 | 0.002843725 | 0.129570038 | -1.6651237 |
| MAP4K3 | -0.502615824 | 6.795701277 | -3.052241745 | 0.003994896 | 0.148337431 | -1.958511171 |
| MXI1 | -0.502697428 | 10.91699694 | -2.208633681 | 0.032905173 | 0.35330217 | -3.737249515 |
| PKIG | -0.502829865 | 7.60254012 | -3.106608812 | 0.003446952 | 0.142973561 | -1.831326586 |
| CPQ | -0.505494405 | 7.886975183 | -2.508210989 | 0.016226927 | 0.262224328 | -3.151258666 |
| MTHFD2L | -0.507167862 | 6.070953433 | -2.508525474 | 0.016214471 | 0.262224328 | -3.150615109 |
| EPHA1 | -0.509102393 | 6.89349195 | -2.332090535 | 0.024738002 | 0.315314181 | -3.502456864 |
| NGRN | -0.51029378 | 10.55829422 | -4.194466653 | 0.00014449 | 0.04717696 | 0.941196477 |
| ARID5A | -0.511225454 | 8.95612829 | -2.955532484 | 0.005177376 | 0.166346672 | -2.181412503 |
| PHOX2B | -0.511785709 | 6.513805313 | -2.0965272 | 0.042310436 | 0.383297321 | -3.941908762 |
| C8orf33 | -0.515809909 | 9.08542686 | -2.929420451 | 0.005548889 | 0.171219895 | -2.240842916 |
| AGAP1 | -0.51921929 | 7.430728384 | -2.061373054 | 0.045710564 | 0.39671268 | -4.004357218 |
| SIAH2 | -0.520502149 | 9.600533577 | -2.420545072 | 0.020058889 | 0.289942129 | -3.328396544 |
| TMEM243 | -0.520891074 | 10.75465972 | -3.446904705 | 0.00133275 | 0.094383859 | -1.007050249 |
| MAST4 | -0.52285102 | 7.611078612 | -2.830624404 | 0.007192115 | 0.192538241 | -2.462693934 |
| BCL11B | -0.523260319 | 11.87891188 | -3.706800394 | 0.000627184 | 0.071591261 | -0.348506661 |
| ZNF571 | -0.523879296 | 7.92010427 | -2.302134788 | 0.026532214 | 0.323870586 | -3.560311972 |
| RPS9 | -0.524766855 | 13.8234747 | -3.686533294 | 0.000665678 | 0.073059677 | -0.400663918 |
| NFKBIA | -0.525682992 | 12.80608327 | -2.273619398 | 0.028347565 | 0.332894222 | -3.614862731 |
| ATOH1 | -0.526459716 | 4.542870901 | -2.062763274 | 0.045571696 | 0.395808933 | -4.001903531 |
| RNF138 | -0.527494355 | 11.64184459 | -3.292026586 | 0.002065252 | 0.112062639 | -1.387975068 |
| DHRS3 | -0.529340608 | 8.806765434 | -2.084159501 | 0.043480482 | 0.388785102 | -3.963974354 |
| CEBPD | -0.53042624 | 8.044428045 | -2.516498713 | 0.015901571 | 0.261781659 | -3.134279691 |
| CLDN15 | -0.531052892 | 8.162440481 | -2.788444002 | 0.008023258 | 0.199693849 | -2.555924966 |
| ZNF329 | -0.531431208 | 8.169049534 | -2.158797488 | 0.036829692 | 0.364513125 | -3.82925412 |
| MIP | -0.53179131 | 6.798768882 | -2.184720891 | 0.034739433 | 0.357245809 | -3.781598514 |
| ZNF37BP | -0.532954628 | 6.448239668 | -2.089566991 | 0.042965463 | 0.386532596 | -3.954339386 |
| FAM169A | -0.534082044 | 7.251959889 | -2.180692909 | 0.035057164 | 0.359024726 | -3.789032171 |
| FOSL2 | -0.534292978 | 8.743434899 | -2.169058005 | 0.035989429 | 0.360242859 | -3.81044474 |
| TRIM28 | -0.535030951 | 11.07386286 | -3.14290558 | 0.003121484 | 0.135833859 | -1.745686771 |
| ARRB1 | -0.53704815 | 8.585659401 | -3.773443193 | 0.000515166 | 0.06839991 | -0.176109644 |
| CAMK2N1 | -0.537159398 | 8.748410091 | -2.662958319 | 0.011052002 | 0.228899115 | -2.827807511 |
| NAMPT | -0.538335985 | 9.058224071 | -3.421388471 | 0.001433343 | 0.09781583 | -1.070433299 |
| NEO1 | -0.541958377 | 7.544923265 | -2.796937527 | 0.007849043 | 0.199693849 | -2.537224799 |
| ZCCHC14 | -0.54243619 | 6.584342748 | -2.60873183 | 0.012661837 | 0.240304388 | -2.942676163 |
| TNFSF4 | -0.544275424 | 8.2645802 | -2.791641553 | 0.007957254 | 0.199693849 | -2.548889275 |
| AGBL5 | -0.54528996 | 5.390369174 | -2.040130314 | 0.047878937 | 0.404767947 | -4.04168574 |
| MAFF | -0.545685704 | 9.790584499 | -2.110688113 | 0.041004747 | 0.378160103 | -3.916517374 |
| NREP | -0.546729532 | 7.720021379 | -4.199372424 | 0.000142328 | 0.04717696 | 0.954468918 |
| USP36 | -0.547524901 | 8.444304426 | -2.974039111 | 0.004928324 | 0.162124851 | -2.139095185 |
| NSG1 | -0.548465858 | 8.934436563 | -2.556022427 | 0.014430313 | 0.253299578 | -3.052764605 |
| LINC00963 | -0.548682947 | 6.019174904 | -2.516142121 | 0.015915447 | 0.261781659 | -3.135011054 |
| IDO1 | -0.550310699 | 7.558606271 | -2.375090683 | 0.02235287 | 0.303867692 | -3.418437482 |
| PCSK5 | -0.551279533 | 7.462337858 | -2.351255692 | 0.023648033 | 0.309696837 | -3.465149963 |
| CTTN | -0.556223581 | 7.040435909 | -2.105028669 | 0.041522261 | 0.378729629 | -3.926681266 |
| AMIGO2 | -0.55636492 | 9.855173173 | -3.933929652 | 0.000319064 | 0.062905236 | 0.244361072 |
| NOL10 | -0.56258594 | 5.537703198 | -2.063634119 | 0.045484895 | 0.395808933 | -4.000365852 |
| TGFBR2 | -0.564726056 | 10.44574577 | -4.545763702 | 4.85E-05 | 0.036034382 | 1.903531528 |
| SORL1 | -0.566727835 | 11.83822476 | -2.427724087 | 0.019716724 | 0.287340547 | -3.314061738 |
| GLI3 | -0.567718392 | 6.780540252 | -2.170413914 | 0.035879668 | 0.35972485 | -3.807953935 |
| IKZF5 | -0.568699155 | 9.40566511 | -3.345719774 | 0.00177608 | 0.103900666 | -1.256960476 |
| SEMA4C | -0.572471528 | 8.966003804 | -2.574150148 | 0.013798078 | 0.247533015 | -3.015079577 |
| RPL10L | -0.572805243 | 8.574999204 | -2.236335709 | 0.030888183 | 0.341392724 | -3.685409745 |
| ZBTB16 | -0.572943947 | 8.237966922 | -3.397792313 | 0.001532792 | 0.10040969 | -1.128831661 |
| SIAH1 | -0.574354039 | 8.95042797 | -4.053947364 | 0.000221972 | 0.053978361 | 0.563324711 |
| ACVRL1 | -0.574527605 | 5.871254545 | -2.115944284 | 0.040529194 | 0.378160103 | -3.907058498 |
| ERO1B | -0.579819574 | 6.182034907 | -2.232489256 | 0.0311615 | 0.342690692 | -3.692637307 |
| BMPR1B | -0.581888171 | 4.775702302 | -2.284286089 | 0.027655891 | 0.330750589 | -3.594517056 |
| ACSBG1 | -0.587143401 | 5.98411334 | -2.324322719 | 0.025192482 | 0.317517644 | -3.517512933 |
| ACPP | -0.588057062 | 5.822363555 | -2.025537303 | 0.049419997 | 0.409423382 | -4.06714988 |
| PYGL | -0.588082727 | 7.080097692 | -2.785556882 | 0.00808329 | 0.199693849 | -2.562273088 |
| PCDHB6 | -0.589665155 | 5.524606133 | -2.106391707 | 0.041397099 | 0.378160103 | -3.924235326 |
| CCR7 | -0.589830333 | 11.41927232 | -2.150416995 | 0.037528803 | 0.367639981 | -3.844565576 |
| PLEKHO1 | -0.59544892 | 8.40507179 | -2.201072103 | 0.033475678 | 0.354750958 | -3.751313544 |
| CLEC11A | -0.599269205 | 7.268784933 | -3.513950661 | 0.001099612 | 0.089756425 | -0.839379869 |
| LIN7B | -0.601827777 | 6.790723166 | -2.122906396 | 0.039906765 | 0.37665426 | -3.894501202 |
| CYP2R1 | -0.60203094 | 6.930999721 | -2.630998819 | 0.01197636 | 0.235763205 | -2.895703439 |
| PRKCA | -0.604843182 | 8.308558549 | -2.490005925 | 0.01696301 | 0.267539204 | -3.188415301 |
| S1PR1 | -0.609634929 | 11.06857322 | -3.372781952 | 0.001645381 | 0.101846663 | -1.190501075 |
| PELI2 | -0.612618215 | 7.838945479 | -2.071120637 | 0.044744626 | 0.39300485 | -3.987125383 |
| NR4A2 | -0.615267804 | 12.13913304 | -2.324322448 | 0.025192498 | 0.317517644 | -3.517513457 |
| JOSD1 | -0.616595593 | 11.10563585 | -3.781957677 | 0.00050233 | 0.06839991 | -0.153987183 |
| PLEKHB1 | -0.616614595 | 9.121598378 | -2.43823256 | 0.019225396 | 0.284688631 | -3.29302324 |
| PROX1 | -0.61747847 | 6.242329667 | -2.312683798 | 0.025887488 | 0.320735893 | -3.540001964 |
| LRP12 | -0.618345261 | 4.504244103 | -2.193574148 | 0.034050029 | 0.356332469 | -3.765222546 |
| CNN3 | -0.619579984 | 6.679873592 | -2.362078464 | 0.023051788 | 0.307234539 | -3.443982351 |
| COL6A2 | -0.61983148 | 8.180182335 | -2.306248319 | 0.026279114 | 0.322238011 | -3.552400472 |
| PLK3 | -0.621964284 | 9.5900535 | -2.897289668 | 0.006040267 | 0.177095475 | -2.313520715 |
| ZNF268 | -0.624298598 | 6.758129413 | -2.338608966 | 0.024362319 | 0.314403202 | -3.489793558 |
| PFKFB3 | -0.624380772 | 9.182267232 | -2.974171387 | 0.004926585 | 0.162124851 | -2.138792139 |
| NBEA | -0.625237717 | 6.705560917 | -3.502622101 | 0.00113605 | 0.090315937 | -0.867823368 |
| ZFP36 | -0.626689425 | 12.97510467 | -3.286315575 | 0.00209852 | 0.112180348 | -1.401843061 |
| AKR1B10 | -0.628116399 | 6.334544841 | -2.043054882 | 0.047575183 | 0.403299672 | -4.036564909 |
| CTSA | -0.629527954 | 9.951912292 | -2.385890954 | 0.021787335 | 0.302244438 | -3.397156509 |
| CSF2RB | -0.632225045 | 8.068484047 | -2.434195993 | 0.0194128 | 0.285551723 | -3.301112446 |
| HGD | -0.636531499 | 6.365999231 | -2.298879084 | 0.026734078 | 0.325373933 | -3.566566102 |
| SC5D | -0.637460806 | 8.668166285 | -3.538153954 | 0.001025481 | 0.08771047 | -0.778459988 |
| GSTM1 | -0.637628462 | 9.226010572 | -3.528121474 | 0.001055608 | 0.088457098 | -0.803736619 |
| S100A3 | -0.63938584 | 5.273290569 | -2.4926036 | 0.016856152 | 0.266985948 | -3.183125228 |
| EPB41L3 | -0.639890708 | 5.306065897 | -2.178137791 | 0.035260045 | 0.359181911 | -3.793742147 |
| TNNC2 | -0.641146015 | 6.658994539 | -2.020319585 | 0.049981384 | 0.410509351 | -4.076218976 |
| PDE4B | -0.643115269 | 10.79333843 | -2.994386987 | 0.004667482 | 0.160370043 | -2.092381344 |
| APBA2 | -0.645015216 | 9.446077 | -2.771689641 | 0.008377487 | 0.202663282 | -2.592704088 |
| HSD17B3 | -0.645095331 | 7.068886289 | -2.639013777 | 0.011738092 | 0.234799699 | -2.878728727 |
| ZNF91 | -0.649461304 | 11.02778801 | -3.32275868 | 0.001894676 | 0.108122832 | -1.313125782 |
| MPL | -0.651046716 | 5.537860139 | -2.252552142 | 0.029759218 | 0.338052043 | -3.654834478 |
| SCRN1 | -0.654427552 | 7.46006927 | -2.447210198 | 0.018814463 | 0.282148698 | -3.274997449 |
| PCSK1N | -0.655540643 | 8.07018612 | -2.349906544 | 0.023723325 | 0.30970176 | -3.467783633 |
| CKAP4 | -0.658847422 | 9.177358631 | -2.740369699 | 0.00907886 | 0.213922091 | -2.661066616 |
| HPGD | -0.658884136 | 7.259800879 | -2.72048647 | 0.009551931 | 0.217913378 | -2.704199055 |
| KRT1 | -0.664491976 | 8.000959071 | -2.714473521 | 0.009699421 | 0.219283809 | -2.71720169 |
| HOXB8 | -0.664875682 | 4.732668424 | -2.083147636 | 0.043577452 | 0.389048124 | -3.965775082 |
| PLXDC1 | -0.66597985 | 8.270889961 | -4.129925434 | 0.000176088 | 0.051996414 | 0.76707577 |
| ZNF395 | -0.668307461 | 9.398917909 | -2.631141308 | 0.011972086 | 0.235763205 | -2.895401973 |
| ANKH | -0.668423781 | 6.805974181 | -2.233670689 | 0.031077323 | 0.342596407 | -3.690418382 |
| RNF130 | -0.669442562 | 7.971931358 | -4.461832854 | 6.31E-05 | 0.039114405 | 1.67158743 |
| EMP2 | -0.670244494 | 6.786932701 | -2.619756075 | 0.012318081 | 0.238718485 | -2.919454561 |
| SCML2 | -0.67086055 | 8.598168134 | -3.306432086 | 0.001983552 | 0.110982008 | -1.352936501 |
| CUX1 | -0.674330547 | 8.051624685 | -4.246233842 | 0.00012321 | 0.04717696 | 1.081510105 |
| MINDY1 | -0.674783687 | 8.8060443 | -2.081885042 | 0.043698717 | 0.389612856 | -3.96802104 |
| PIK3IP1 | -0.674841783 | 11.98342326 | -3.306642133 | 0.001982384 | 0.110982008 | -1.352424991 |
| ZFP30 | -0.675980776 | 6.505376495 | -2.080438664 | 0.043837995 | 0.389949437 | -3.970592594 |
| STARD5 | -0.676068636 | 7.381512516 | -3.487481014 | 0.00118656 | 0.091489527 | -0.905768281 |
| AHDC1 | -0.67736601 | 7.327322059 | -2.739140216 | 0.009107472 | 0.213922091 | -2.663739763 |
| ZSCAN18 | -0.678757734 | 8.462465909 | -3.129940185 | 0.003234258 | 0.13877021 | -1.776343821 |
| ZBTB18 | -0.684252232 | 9.392465866 | -2.643372573 | 0.011610351 | 0.233831881 | -2.869482511 |
| URGCP | -0.684673533 | 8.272348644 | -2.893179389 | 0.00610598 | 0.177249618 | -2.32278164 |
| PMCHL1 | -0.685292661 | 3.514964552 | -2.031810261 | 0.048752344 | 0.406335061 | -4.056221798 |
| TMEM8B | -0.686664893 | 7.496415133 | -2.727769992 | 0.009376053 | 0.216539676 | -2.688423224 |
| ACTN1 | -0.687074187 | 9.98554965 | -2.454269306 | 0.01849696 | 0.279666262 | -3.26079019 |
| IFT81 | -0.695371498 | 5.037037861 | -2.270008271 | 0.028585204 | 0.332919268 | -3.621734289 |
| SKIL | -0.69642414 | 7.718611495 | -3.250786814 | 0.002317206 | 0.11634811 | -1.487821784 |
| PFN2 | -0.702167661 | 8.668624913 | -3.503447128 | 0.001133357 | 0.090315937 | -0.865753438 |
| NAP1L3 | -0.704363087 | 8.626553291 | -2.535775674 | 0.015167694 | 0.258293855 | -3.094634005 |
| ARMCX1 | -0.70487532 | 6.282660126 | -2.849622453 | 0.006844575 | 0.188110706 | -2.420408476 |
| JAM3 | -0.706936812 | 8.519955556 | -3.149836933 | 0.00306273 | 0.134219019 | -1.729267491 |
| RNFT2 | -0.709875443 | 5.137123719 | -2.737102103 | 0.009155085 | 0.214218213 | -2.668169283 |
| SMIM14 | -0.710337377 | 4.924439035 | -2.175962753 | 0.035433565 | 0.359181911 | -3.797748133 |
| SNN | -0.71197577 | 9.858905576 | -3.284239241 | 0.00211074 | 0.112349371 | -1.406881762 |
| ARHGAP32 | -0.719690181 | 6.347064258 | -2.176514432 | 0.035389482 | 0.359181911 | -3.796732344 |
| CPA3 | -0.719691241 | 7.841863761 | -2.609743467 | 0.01262993 | 0.240304388 | -2.940548048 |
| BACH2 | -0.723477846 | 8.625397542 | -3.27294758 | 0.002178394 | 0.113271021 | -1.434253215 |
| MMP28 | -0.730949187 | 3.973642655 | -2.199769586 | 0.033574831 | 0.354750958 | -3.753732389 |
| CD1B | -0.732354937 | 5.279174221 | -2.262828313 | 0.029062992 | 0.33623062 | -3.635372341 |
| UNC119B | -0.734679501 | 8.987823245 | -3.755309717 | 0.00054357 | 0.06839991 | -0.223152239 |
| RAB20 | -0.73824316 | 7.489863243 | -2.578076916 | 0.013664512 | 0.247533015 | -3.006891898 |
| ADRA1B | -0.739129451 | 4.751356315 | -2.639994413 | 0.011709241 | 0.234600974 | -2.876649435 |
| GK3P | -0.741083523 | 5.844236977 | -2.03421138 | 0.048498871 | 0.405283446 | -4.052031658 |
| KLRB1 | -0.742745784 | 11.88249342 | -2.922891355 | 0.005645606 | 0.173067232 | -2.255651826 |
| ID1 | -0.744274301 | 7.668585246 | -2.625126399 | 0.012153749 | 0.238121319 | -2.908118062 |
| WDR19 | -0.744348109 | 7.398733804 | -2.110040027 | 0.04106372 | 0.378160103 | -3.917682372 |
| MGMT | -0.744364606 | 8.13380192 | -2.336320686 | 0.024493613 | 0.315113893 | -3.49424198 |
| CLEC1B | -0.745189739 | 8.380701832 | -2.668512573 | 0.010898225 | 0.227754622 | -2.815951031 |
| GSTM2 | -0.747733065 | 9.595905798 | -3.706395905 | 0.000627931 | 0.071591261 | -0.349548866 |
| GCM1 | -0.750740053 | 5.303552221 | -2.327960839 | 0.024978697 | 0.317078614 | -3.510465965 |
| RAI2 | -0.758125889 | 5.985622331 | -2.412842346 | 0.020431985 | 0.291596641 | -3.343742707 |
| IGFBP6 | -0.759741374 | 6.212941286 | -2.023730224 | 0.049613801 | 0.409556494 | -4.070292951 |
| MYO15B | -0.760574125 | 8.818079347 | -4.221108479 | 0.000133125 | 0.04717696 | 1.013337488 |
| TCEAL2 | -0.760738274 | 6.042410427 | -2.558583353 | 0.014339417 | 0.253007896 | -3.047452087 |
| ATP1B1 | -0.760932111 | 7.390701938 | -2.83851206 | 0.007045874 | 0.191209921 | -2.445159769 |
| IGF1R | -0.767145135 | 7.339814458 | -3.449646864 | 0.00132235 | 0.094383859 | -1.000224407 |
| SSBP2 | -0.767615874 | 7.907146666 | -2.637117885 | 0.011794056 | 0.235160578 | -2.882747186 |
| ITM2C | -0.76806259 | 8.625399674 | -2.238180143 | 0.030757881 | 0.341313226 | -3.681940648 |
| SLC2A3 | -0.771667657 | 11.5645731 | -3.04007343 | 0.004128296 | 0.151926195 | -1.986795009 |
| PPP2R2D | -0.774090441 | 7.20018552 | -2.251296211 | 0.029845321 | 0.338052043 | -3.657208482 |
| ABCC3 | -0.775601989 | 5.267242371 | -2.047813852 | 0.047084494 | 0.401610657 | -4.028219567 |
| DCHS1 | -0.780038419 | 7.065498101 | -3.266266493 | 0.002219389 | 0.113271021 | -1.450424214 |
| HDC | -0.786747326 | 6.683325893 | -2.110166761 | 0.041052182 | 0.378160103 | -3.917454577 |
| RGCC | -0.789200529 | 11.75905804 | -2.188337595 | 0.03445632 | 0.356332469 | -3.774914837 |
| PBX1 | -0.789987938 | 6.73788559 | -2.374506239 | 0.022383847 | 0.303867692 | -3.419587049 |
| VIPR1 | -0.791645945 | 8.9847566 | -2.619095226 | 0.012338444 | 0.238722903 | -2.920848478 |
| MAP3K7CL | -0.795490652 | 8.514754432 | -2.089296555 | 0.042991092 | 0.386532596 | -3.954821713 |
| EHD3 | -0.798002676 | 7.537428519 | -2.192892647 | 0.034102664 | 0.356332469 | -3.766484943 |
| MEIS1 | -0.800302593 | 7.171754067 | -2.501711955 | 0.016486294 | 0.262805935 | -3.164545337 |
| RAB3A | -0.803272075 | 7.180735471 | -2.271899045 | 0.028460556 | 0.332919268 | -3.618137395 |
| DOK3 | -0.803430818 | 5.663829081 | -2.139079782 | 0.038493195 | 0.370934423 | -3.865205121 |
| NT5E | -0.803875142 | 7.146686347 | -2.083575359 | 0.043536439 | 0.389004984 | -3.965013985 |
| TSC22D1 | -0.803939951 | 10.13697179 | -2.893349606 | 0.006103245 | 0.177249618 | -2.322398285 |
| FGF9 | -0.816284401 | 8.081283734 | -3.46318391 | 0.001272139 | 0.092806307 | -0.966487517 |
| MPIG6B | -0.816425244 | 6.589346796 | -2.696206318 | 0.010160501 | 0.223546489 | -2.756585339 |
| SEC14L2 | -0.823163787 | 7.545856822 | -2.102250121 | 0.041778431 | 0.379691229 | -3.931663436 |
| PDGFRL | -0.824098676 | 4.778644553 | -2.156598962 | 0.037011974 | 0.364513125 | -3.833275392 |
| SLC19A2 | -0.825312488 | 6.595624145 | -3.531741125 | 0.001044642 | 0.088133661 | -0.794621017 |
| GADD45G | -0.828398217 | 7.158679123 | -2.536263122 | 0.015149544 | 0.258293855 | -3.093628735 |
| PTPRO | -0.829982248 | 10.29798108 | -3.772161794 | 0.000517125 | 0.06839991 | -0.179437128 |
| KLHL21 | -0.833678778 | 7.882831369 | -2.279703927 | 0.027951147 | 0.331902675 | -3.603265895 |
| ZNF10 | -0.837858237 | 7.067595814 | -2.513071982 | 0.016035368 | 0.262224328 | -3.141304815 |
| RGS16 | -0.838663181 | 7.49529951 | -3.823749553 | 0.000443677 | 0.06737744 | -0.045094143 |
| TGIF1 | -0.843188166 | 8.934388981 | -3.555074196 | 0.000976531 | 0.086366788 | -0.735750898 |
| GPHN | -0.850883748 | 6.107911252 | -2.651268467 | 0.011382204 | 0.23103452 | -2.852706635 |
| SGK1 | -0.861338694 | 10.33551037 | -2.245361603 | 0.030255193 | 0.339570052 | -3.668412645 |
| ENO3 | -0.86360812 | 6.661667085 | -2.706763088 | 0.009891629 | 0.221404475 | -2.733846907 |
| IRS2 | -0.864701018 | 11.44104523 | -2.739878176 | 0.009090288 | 0.213922091 | -2.662135382 |
| RAB31 | -0.865089241 | 7.648208981 | -2.269952953 | 0.028588858 | 0.332919268 | -3.621839489 |
| PDGFC | -0.871175663 | 7.024453573 | -2.087024951 | 0.043206901 | 0.386903366 | -3.958871206 |
| MEST | -0.875711324 | 8.522154157 | -4.203064664 | 0.000140722 | 0.04717696 | 0.96446161 |
| KCNQ1 | -0.875954578 | 7.348416542 | -2.240642249 | 0.030584707 | 0.341107496 | -3.677306403 |
| NRIP1 | -0.88022244 | 9.343271873 | -3.215349711 | 0.002556771 | 0.123284731 | -1.573064514 |
| KLF11 | -0.886574921 | 9.073690946 | -4.536851683 | 4.99E-05 | 0.036034382 | 1.878849133 |
| SCML1 | -0.896725843 | 7.8707692 | -2.340397644 | 0.024260131 | 0.313410563 | -3.48631411 |
| KRT5 | -0.898914308 | 6.408985585 | -2.577725778 | 0.013676408 | 0.247533015 | -3.007624407 |
| ATP9A | -0.901142443 | 5.109439889 | -2.282566691 | 0.027766354 | 0.331432453 | -3.5978015 |
| MFAP3L | -0.90447185 | 6.448985677 | -2.71949527 | 0.0095761 | 0.217913378 | -2.706343784 |
| LTBP3 | -0.906379511 | 9.040495083 | -2.969203152 | 0.004992296 | 0.162478656 | -2.150168749 |
| MMD | -0.916769631 | 10.43876765 | -2.828304754 | 0.007235657 | 0.192981979 | -2.467844521 |
| CLN6 | -0.925279815 | 5.693346035 | -3.014829968 | 0.004418514 | 0.155677319 | -2.045254542 |
| MXD1 | -0.927304689 | 6.024033889 | -2.779856562 | 0.008203044 | 0.200463344 | -2.57479419 |
| NELL2 | -0.940050686 | 11.39308654 | -3.672527488 | 0.000693601 | 0.074221565 | -0.436632174 |
| CLC | -0.948430797 | 7.88836949 | -2.443281918 | 0.018993282 | 0.283395462 | -3.282890759 |
| RPS8 | -0.967151315 | 4.767420019 | -2.936407765 | 0.005447103 | 0.169310696 | -2.224971959 |
| KLHL26 | -0.96965201 | 6.932967371 | -2.899774344 | 0.006000864 | 0.176776035 | -2.307918451 |
| EFHC1 | -0.972807472 | 4.490119269 | -2.519802027 | 0.015773558 | 0.26083155 | -3.127501164 |
| GFI1B | -0.976961679 | 6.513110649 | -2.998807819 | 0.004612551 | 0.159949701 | -2.082206552 |
| AREG | -0.97955281 | 8.491172135 | -2.706111275 | 0.009908038 | 0.221404475 | -2.735252584 |
| NR3C2 | -0.981116635 | 8.788132622 | -3.239081148 | 0.002393878 | 0.119232437 | -1.516036642 |
| H2AC6 | -0.983561882 | 10.19455859 | -2.064655977 | 0.045383227 | 0.395254758 | -3.998560867 |
| CLIP2 | -0.987148236 | 5.949040564 | -2.316747721 | 0.025642885 | 0.320673337 | -3.532159138 |
| TPM2 | -0.995350162 | 5.488138837 | -2.172635883 | 0.035700438 | 0.359672489 | -3.803869564 |
| ZNF331 | -0.997644207 | 11.33666234 | -2.749918227 | 0.008859472 | 0.210892838 | -2.640279177 |
| ADGRA3 | -1.009455393 | 4.796853255 | -2.683055695 | 0.010504872 | 0.225011094 | -2.784827193 |
| SMAD1 | -1.013196766 | 5.537587355 | -3.554818889 | 0.000977253 | 0.086366788 | -0.736396059 |
| ZNF165 | -1.02636543 | 4.659418827 | -2.180036721 | 0.035109167 | 0.359181911 | -3.790242163 |
| GAS2L1 | -1.027564145 | 6.814595886 | -2.714753476 | 0.009692507 | 0.219283809 | -2.716596729 |
| PDK1 | -1.029426851 | 7.296431486 | -2.437995891 | 0.019236339 | 0.284688631 | -3.293497787 |
| ROBO1 | -1.039212388 | 5.026759197 | -2.12530729 | 0.039694078 | 0.375504162 | -3.890163293 |
| GP9 | -1.046638256 | 7.631873405 | -3.275259363 | 0.002164377 | 0.113259951 | -1.428653541 |
| MEGF6 | -1.058056754 | 6.729402589 | -3.140920859 | 0.003138503 | 0.136096914 | -1.750384432 |
| TCEA2 | -1.071514953 | 7.060644628 | -2.833607565 | 0.007136477 | 0.192243187 | -2.456066087 |
| EREG | -1.110895209 | 5.235251487 | -3.291342543 | 0.00206921 | 0.112062639 | -1.389636812 |
| GADD45A | -1.143663056 | 11.27176841 | -4.122020304 | 0.000180394 | 0.052029039 | 0.745813603 |
| PLK2 | -1.147615812 | 7.0952939 | -2.994338753 | 0.004668085 | 0.160370043 | -2.092492309 |
| TNS3 | -1.148073774 | 4.03577905 | -2.854859688 | 0.006751556 | 0.187321692 | -2.408719743 |
| SOX4 | -1.151051455 | 7.455399646 | -4.083791181 | 0.000202707 | 0.053978361 | 0.643194544 |
| GRIN2D | -1.159967495 | 4.605147663 | -3.35547596 | 0.00172786 | 0.101846663 | -1.233033723 |
| F13A1 | -1.17996829 | 9.482158131 | -2.15168773 | 0.037422049 | 0.366883994 | -3.842246884 |
| CA2 | -1.193342847 | 7.297587194 | -2.183552778 | 0.034831311 | 0.357597615 | -3.783755364 |
| CFD | -1.206853665 | 5.168881731 | -2.359565449 | 0.023189015 | 0.308142575 | -3.44890382 |
| FCER1A | -1.211135144 | 7.720501481 | -2.055150118 | 0.046336714 | 0.39876975 | -4.015324322 |
| DSC1 | -1.235571657 | 6.490442978 | -2.463722977 | 0.018079371 | 0.277260134 | -3.241717418 |
| SPINK2 | -1.25447771 | 6.759123231 | -3.846694627 | 0.000414352 | 0.065881921 | 0.014906113 |
| DPEP2 | -1.258654324 | 8.177084368 | -2.381189929 | 0.022031889 | 0.302579663 | -3.406428189 |
| CDKN1A | -1.259648277 | 9.100371733 | -3.360434212 | 0.001703835 | 0.101846663 | -1.22085967 |
| FBLN5 | -1.302416433 | 7.594040347 | -2.520951986 | 0.015729214 | 0.26083155 | -3.125139928 |
| MATN3 | -1.333667928 | 2.748311661 | -3.038719191 | 0.004143399 | 0.151964702 | -1.989938609 |
| CD9 | -1.379710699 | 7.05327136 | -2.219915393 | 0.032070039 | 0.347668377 | -3.71619742 |
| SMAD7 | -1.391190231 | 10.05577448 | -4.245006894 | 0.000123677 | 0.04717696 | 1.078177963 |
| CRIP2 | -1.482994089 | 7.340764178 | -2.889149856 | 0.00617105 | 0.177571608 | -2.331852596 |
| PF4 | -1.54665218 | 10.53339572 | -2.269020809 | 0.028650496 | 0.333324058 | -3.623611875 |
| PRKAR2B | -1.606150067 | 8.64133817 | -2.238032717 | 0.030768278 | 0.341313226 | -3.682218013 |
| C5AR1 | -1.610907907 | 4.777214447 | -3.430444306 | 0.001396838 | 0.09677981 | -1.047965847 |
| LYPD3 | -1.633632909 | 8.789769664 | -3.234537752 | 0.002424281 | 0.12026375 | -1.526972688 |
| NRGN | -1.802396406 | 9.32392703 | -2.248880872 | 0.030011535 | 0.338366411 | -3.661771205 |

Supplementary Table 2. Clue GO Result

| ID | Term | Ontology Source | Term PValue | Term P Value Corrected with Bonferroni step down | Group PValue | Group P Value Corrected with Bonferroni step down | GOLevels | GOGroups | % Associated Genes | Nr. Genes | Associated Genes Found |
| --- | --- | --- | --- | --- | --- | --- | --- | --- | --- | --- | --- |
| GO:0061980 | regulatory RNA binding | GO_MolecularFunction-EBI-UniProt-GOA-ACAP-ARAP_13.05.2021_00h00 | 0.01 | 0.01 | 0.01 | 0.04 | [5] | Group0 | 2.00 | 1.00 | [DPEP2] |
| GO:0004402 | histone acetyltransferase activity | GO_BiologicalProcess-EBI-UniProt-GOA-ACAP-ARAP_13.05.2021_00h00 | 0.01 | 0.04 | 0.04 | 0.04 | [8, 9, 10, 11, 12] | Group1 | 2.08 | 1.00 | [ARRB1] |
| GO:0022602 | ovulation cycle process | GO_BiologicalProcess-EBI-UniProt-GOA-ACAP-ARAP_13.05.2021_00h00 | 0.01 | 0.02 | 0.04 | 0.04 | [2, 3, 4, 5] | Group1 | 2.04 | 1.00 | [ARRB1] |
| GO:0031762 | follicle-stimulating hormone receptor binding | GO_MolecularFunction-EBI-UniProt-GOA-ACAP-ARAP_13.05.2021_00h00 | 0.00 | 0.02 | 0.04 | 0.04 | [5] | Group1 | 50.00 | 1.00 | [ARRB1] |
| GO:0061733 | peptide-lysine-N-acetyltransferase activity | GO_MolecularFunction-EBI-UniProt-GOA-ACAP-ARAP_13.05.2021_00h00 | 0.01 | 0.01 | 0.04 | 0.04 | [3, 8] | Group1 | 2.00 | 1.00 | [ARRB1] |
| GO:0031691 | alpha-1A adrenergic receptor binding | GO_MolecularFunction-EBI-UniProt-GOA-ACAP-ARAP_13.05.2021_00h00 | 0.00 | 0.02 | 0.04 | 0.04 | [6] | Group1 | 50.00 | 1.00 | [ARRB1] |
| GO:0031692 | alpha-1B adrenergic receptor binding | GO_MolecularFunction-EBI-UniProt-GOA-ACAP-ARAP_13.05.2021_00h00 | 0.00 | 0.04 | 0.04 | 0.04 | [6] | Group1 | 33.33 | 1.00 | [ARRB1] |
| GO:0031896 | V2 vasopressin receptor binding | GO_MolecularFunction-EBI-UniProt-GOA-ACAP-ARAP_13.05.2021_00h00 | 0.00 | 0.01 | 0.04 | 0.04 | [6] | Group1 | 100.00 | 1.00 | [ARRB1] |
| GO:0032715 | negative regulation of interleukin-6 production | GO_BiologicalProcess-EBI-UniProt-GOA-ACAP-ARAP_13.05.2021_00h00 | 0.01 | 0.05 | 0.04 | 0.04 | [4, 5, 6, 7, 8] | Group1 | 2.13 | 1.00 | [ARRB1] |
| GO:0042699 | follicle-stimulating hormone signaling pathway | GO_BiologicalProcess-EBI-UniProt-GOA-ACAP-ARAP_13.05.2021_00h00 | 0.00 | 0.05 | 0.04 | 0.04 | [3, 4, 5, 6, 7, 8, 9, 10] | Group1 | 25.00 | 1.00 | [ARRB1] |
| GO:0050436 | microfibril binding | GO_MolecularFunction-EBI-UniProt-GOA-ACAP-ARAP_13.05.2021_00h00 | 0.00 | 0.05 | 0.00 | 0.01 | [2] | Group2 | 25.00 | 1.00 | [LTBP3] |
| GO:0032964 | collagen biosynthetic process | GO_BiologicalProcess-EBI-UniProt-GOA-ACAP-ARAP_13.05.2021_00h00 | 0.01 | 0.01 | 0.00 | 0.01 | [3] | Group2 | 2.00 | 1.00 | [RGCC] |
| GO:1903053 | regulation of extracellular matrix organization | GO_BiologicalProcess-EBI-UniProt-GOA-ACAP-ARAP_13.05.2021_00h00 | 0.01 | 0.01 | 0.00 | 0.01 | [4, 5, 6] | Group2 | 2.00 | 1.00 | [RGCC] |
| GO:0045124 | regulation of bone resorption | GO_BiologicalProcess-EBI-UniProt-GOA-ACAP-ARAP_13.05.2021_00h00 | 0.01 | 0.01 | 0.00 | 0.01 | [3, 5, 6, 7] | Group2 | 2.00 | 1.00 | [LTBP3] |
| GO:0085029 | extracellular matrix assembly | GO_BiologicalProcess-EBI-UniProt-GOA-ACAP-ARAP_13.05.2021_00h00 | 0.00 | 0.00 | 0.00 | 0.01 | [5, 6] | Group2 | 4.00 | 2.00 | [LTBP3, RGCC] |
| GO:0090272 | negative regulation of fibroblast growth factor production | GO_BiologicalProcess-EBI-UniProt-GOA-ACAP-ARAP_13.05.2021_00h00 | 0.00 | 0.02 | 0.00 | 0.01 | [4, 5, 6, 7, 8] | Group2 | 50.00 | 1.00 | [RGCC] |
| GO:0010718 | positive regulation of epithelial to mesenchymal transition | GO_BiologicalProcess-EBI-UniProt-GOA-ACAP-ARAP_13.05.2021_00h00 | 0.01 | 0.01 | 0.00 | 0.01 | [3, 4, 5, 6, 7, 8, 9, 10] | Group2 | 2.00 | 1.00 | [RGCC] |
| GO:1902808 | positive regulation of cell cycle G1/S phase transition | GO_BiologicalProcess-EBI-UniProt-GOA-ACAP-ARAP_13.05.2021_00h00 | 0.01 | 0.05 | 0.00 | 0.01 | [5, 6, 7, 8] | Group2 | 2.13 | 1.00 | [RGCC] |
| GO:0001100 | negative regulation of exit from mitosis | GO_BiologicalProcess-EBI-UniProt-GOA-ACAP-ARAP_13.05.2021_00h00 | 0.00 | 0.01 | 0.00 | 0.01 | [6, 7, 8, 9, 10] | Group2 | 100.00 | 1.00 | [RGCC] |
| GO:0044467 | glial cell-derived neurotrophic factor production | GO_BiologicalProcess-EBI-UniProt-GOA-ACAP-ARAP_13.05.2021_00h00 | 0.00 | 0.04 | 0.03 | 0.06 | [3, 6] | Group3 | 33.33 | 1.00 | [SORL1] |
| GO:0061502 | early endosome to recycling endosome transport | GO_BiologicalProcess-EBI-UniProt-GOA-ACAP-ARAP_13.05.2021_00h00 | 0.00 | 0.05 | 0.03 | 0.06 | [4, 5, 6] | Group3 | 25.00 | 1.00 | [SORL1] |
| GO:1902769 | regulation of choline O-acetyltransferase activity | GO_BiologicalProcess-EBI-UniProt-GOA-ACAP-ARAP_13.05.2021_00h00 | 0.00 | 0.01 | 0.03 | 0.06 | [5] | Group3 | 100.00 | 1.00 | [SORL1] |
| GO:1900166 | regulation of glial cell-derived neurotrophic factor production | GO_BiologicalProcess-EBI-UniProt-GOA-ACAP-ARAP_13.05.2021_00h00 | 0.00 | 0.04 | 0.03 | 0.06 | [4, 5, 6, 7] | Group3 | 33.33 | 1.00 | [SORL1] |
| GO:1902954 | regulation of early endosome to recycling endosome transport | GO_BiologicalProcess-EBI-UniProt-GOA-ACAP-ARAP_13.05.2021_00h00 | 0.00 | 0.02 | 0.03 | 0.06 | [4, 5, 6, 7] | Group3 | 50.00 | 1.00 | [SORL1] |
| GO:1904179 | positive regulation of adipose tissue development | GO_BiologicalProcess-EBI-UniProt-GOA-ACAP-ARAP_13.05.2021_00h00 | 0.00 | 0.05 | 0.03 | 0.06 | [3, 4, 5, 6, 7, 8] | Group3 | 25.00 | 1.00 | [SORL1] |
| GO:0032527 | protein exit from endoplasmic reticulum | GO_BiologicalProcess-EBI-UniProt-GOA-ACAP-ARAP_13.05.2021_00h00 | 0.01 | 0.02 | 0.03 | 0.06 | [5, 6, 7] | Group3 | 2.04 | 1.00 | [SORL1] |
| GO:1900168 | positive regulation of glial cell-derived neurotrophic factor production | GO_BiologicalProcess-EBI-UniProt-GOA-ACAP-ARAP_13.05.2021_00h00 | 0.00 | 0.04 | 0.03 | 0.06 | [4, 5, 6, 7, 8] | Group3 | 33.33 | 1.00 | [SORL1] |
| GO:1902771 | positive regulation of choline O-acetyltransferase activity | GO_BiologicalProcess-EBI-UniProt-GOA-ACAP-ARAP_13.05.2021_00h00 | 0.00 | 0.01 | 0.03 | 0.06 | [6] | Group3 | 100.00 | 1.00 | [SORL1] |
| GO:1902988 | neurofibrillary tangle assembly | GO_BiologicalProcess-EBI-UniProt-GOA-ACAP-ARAP_13.05.2021_00h00 | 0.00 | 0.04 | 0.03 | 0.06 | [6] | Group3 | 33.33 | 1.00 | [SORL1] |
| GO:1902991 | regulation of amyloid precursor protein catabolic process | GO_BiologicalProcess-EBI-UniProt-GOA-ACAP-ARAP_13.05.2021_00h00 | 0.01 | 0.05 | 0.03 | 0.06 | [5, 6, 7] | Group3 | 2.13 | 1.00 | [SORL1] |
| GO:1902996 | regulation of neurofibrillary tangle assembly | GO_BiologicalProcess-EBI-UniProt-GOA-ACAP-ARAP_13.05.2021_00h00 | 0.00 | 0.02 | 0.03 | 0.06 | [5, 6, 7] | Group3 | 50.00 | 1.00 | [SORL1] |
| GO:0034205 | amyloid-beta formation | GO_BiologicalProcess-EBI-UniProt-GOA-ACAP-ARAP_13.05.2021_00h00 | 0.01 | 0.01 | 0.03 | 0.06 | [6, 7] | Group3 | 2.00 | 1.00 | [SORL1] |
| GO:1902955 | positive regulation of early endosome to recycling endosome transport | GO_BiologicalProcess-EBI-UniProt-GOA-ACAP-ARAP_13.05.2021_00h00 | 0.00 | 0.01 | 0.03 | 0.06 | [5, 6, 7, 8] | Group3 | 100.00 | 1.00 | [SORL1] |
| GO:1902997 | negative regulation of neurofibrillary tangle assembly | GO_BiologicalProcess-EBI-UniProt-GOA-ACAP-ARAP_13.05.2021_00h00 | 0.00 | 0.01 | 0.03 | 0.06 | [5, 6, 7, 8] | Group3 | 100.00 | 1.00 | [SORL1] |
| GO:0004102 | choline O-acetyltransferase activity | GO_MolecularFunction-EBI-UniProt-GOA-ACAP-ARAP_13.05.2021_00h00 | 0.00 | 0.04 | 0.03 | 0.06 | [7] | Group3 | 33.33 | 1.00 | [SORL1] |
| GO:1902962 | regulation of metalloendopeptidase activity involved in amyloid precursor protein catabolic process | GO_BiologicalProcess-EBI-UniProt-GOA-ACAP-ARAP_13.05.2021_00h00 | 0.00 | 0.02 | 0.03 | 0.06 | [6, 7, 8, 9, 10] | Group3 | 50.00 | 1.00 | [SORL1] |
| GO:0038020 | insulin receptor recycling | GO_BiologicalProcess-EBI-UniProt-GOA-ACAP-ARAP_13.05.2021_00h00 | 0.00 | 0.01 | 0.03 | 0.06 | [5, 6, 7, 8, 9, 10, 11, 12] | Group3 | 100.00 | 1.00 | [SORL1] |
| GO:1902960 | negative regulation of aspartic-type endopeptidase activity involved in amyloid precursor protein catabolic process | GO_BiologicalProcess-EBI-UniProt-GOA-ACAP-ARAP_13.05.2021_00h00 | 0.00 | 0.05 | 0.03 | 0.06 | [6, 7, 8, 9, 10, 11] | Group3 | 25.00 | 1.00 | [SORL1] |
| GO:1902963 | negative regulation of metalloendopeptidase activity involved in amyloid precursor protein catabolic process | GO_BiologicalProcess-EBI-UniProt-GOA-ACAP-ARAP_13.05.2021_00h00 | 0.00 | 0.02 | 0.03 | 0.06 | [6, 7, 8, 9, 10, 11, 12] | Group3 | 50.00 | 1.00 | [SORL1] |
| GO:1902948 | negative regulation of tau-protein kinase activity | GO_BiologicalProcess-EBI-UniProt-GOA-ACAP-ARAP_13.05.2021_00h00 | 0.00 | 0.04 | 0.03 | 0.06 | [8, 9, 10, 11] | Group3 | 33.33 | 1.00 | [SORL1] |
| GO:1904684 | negative regulation of metalloendopeptidase activity | GO_BiologicalProcess-EBI-UniProt-GOA-ACAP-ARAP_13.05.2021_00h00 | 0.00 | 0.05 | 0.03 | 0.06 | [8, 9, 10, 11] | Group3 | 25.00 | 1.00 | [SORL1] |

Supplementary Table 3. The potential drugs or molecular compounds

| Genes | Drugs |
| --- | --- |
| DPEP2 | Amphetamine |
| DPEP2 | Nickel |
| DPEP2 | Tobacco Smoke Pollution |
| RGCC | Ethanol |
| RGCC | Methamphetamine |
| RGCC | Nickel |
| RGCC | Tobacco Smoke Pollution |
| LTBP3 | Dronabinol |
| LTBP3 | Ethanol |
| LTBP3 | Tobacco Smoke Pollution |
| ARRB1 | Amphetamine |
| ARRB1 | Dronabinol |
| ARRB1 | Ethanol |
| ARRB1 | JHW 015 |
| ARRB1 | Tobacco Smoke Pollution |
